# Urbanisation Drives the Decoupling, Simplification, and Homogenization of Aquatic and Terrestrial Food Webs

**DOI:** 10.1111/ele.70212

**Published:** 2025-09-14

**Authors:** Kilian Perrelet, Lauren M. Cook, Merin Reji Chacko, Florian Altermatt, Marco Moretti

**Affiliations:** ^1^ Swiss Federal Institute of Aquatic Science and Technology, Eawag Dübendorf Switzerland; ^2^ Department of Evolutionary Biology and Environmental Studies University of Zurich Zurich Switzerland; ^3^ Swiss Federal Research Institute for Forests, Snow, and Landscapes, WSL Birmensdorf Switzerland; ^4^ Department of Environmental Systems Science, Institute of Terrestrial Ecosystems, ETH Zürich Zurich Switzerland

**Keywords:** aquatic‐terrestrial linkages, biodiversity, environmental DNA, metaweb, predators, Urban ecology

## Abstract

Aquatic and terrestrial communities often co‐occur at close distances, enabling biotic interactions across ecosystem boundaries. While such interactions in natural habitats contribute to complex, coupled food webs, their dynamics in engineered and fragmented urban habitats are hardly known. Using environmental DNA metabarcoding and a metaweb approach, we examined food web structure at 54 paired aquatic‐terrestrial sites along an urbanisation gradient in Zurich, Switzerland. We found that urbanisation led to simpler, less connected, and more homogeneous food webs by replacing high‐trophic‐level predators with low‐trophic‐level basal consumers. This shift towards basal consumers, dependent on distinct aquatic or terrestrial basal resources, subsequently weakened the links between aquatic and terrestrial food webs. Conversely, enhancing habitat quantity and landscape connectivity bolstered predator diversity, promoting vertically diverse, connected, complex, and stable food webs. Our findings reveal that while urbanisation can disrupt aquatic‐terrestrial food webs, careful urban habitat planning can enhance biodiversity and food web stability.

## Graphical abstract

Aquatic and terrestrial ecosystems are closely linked, yet how urbanisation affects their interconnected food webs remains poorly understood. To address this gap, we combined environmental DNA metabarcoding with a metaweb approach to analyse food web composition and structure across 54 paired aquatic‐terrestrial sites along an urbanisation gradient in Zurich, Switzerland. We found that urbanisation simplified, homogenised, and decoupled aquatic‐terrestrial food webs by replacing predators with low‐trophic‐level basal consumers, whereas greater habitat quantity and connectivity supported more complex, predator‐rich, and potentially more stable networks.

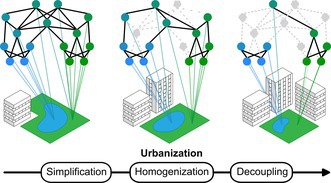

## 1 Introduction

Despite being frequently studied in isolation, aquatic and terrestrial communities are known to interact and establish combined aquatic‐terrestrial food webs (McFadden et al. 2022), in which predators are key in underpinning the functions of both ecosystems and underlying services (e.g., pollination, herbivory, pest control; Knight et al. 2005; Wang and Brose 2018; Shipley et al. 2024). The functionality and stability of these food webs are increasingly linked to environmental gradients (e.g., elevation; Ho et al. 2022), highlighting the need to understand how these gradients influence the composition, structure, and ultimately, the stability of food webs, especially in landscapes facing rapid anthropogenic pressures.

Urban areas, home to nearly 60% of the global human population (United Nations 2018), represent one of the most extreme examples of land‐use conversion, facing escalating pressures from climate change and biodiversity loss (Urban et al. 2024). Although studies have investigated the impact of urbanisation on species richness and community composition (McKinney 2006; Beninde et al. 2015), there is still limited knowledge on how to protect and restore biodiversity in cities. In particular, urban blue and green spaces—often overlapping, such as ponds within parks—have garnered attention for their multifunctional benefits (Cook et al. 2024) and their role in biodiversity conservation, as highlighted in the Global Biodiversity framework (Convention on Biological Diversity 2021). Key strategies include improving habitat quality and quantity at the local scale and enhancing connectivity and complementarity at the landscape scale, both of which can provide additional habitats, resources, and opportunities for species dispersal (Colding 2007; Beninde et al. 2015; Perrelet et al. 2024). However, since most urban habitats are fragmented, isolated, and artificially built—unlike the long‐lasting, co‐evolved aquatic and terrestrial ecosystems found in natural environments—the effects of urbanisation and the efficacy of these conservation strategies on aquatic and terrestrial food webs remain poorly understood.

Increasing urbanisation—as with many other anthropogenic land‐use changes (Newbold et al. 2020; Keck et al. 2025)—typically reduces species richness, particularly among top predators, specialists, and rare species, while favouring generalists (Burkman and Gardiner 2014; Theodorou et al. 2020). This shift in species composition may lead to urban food webs becoming smaller, more connected, less nested, and potentially less stable (i.e., less able to maintain structure and function in the face of disturbances) than those in natural ecosystems (see Table 1 for definitions) (Tylianakis and Morris 2017; Start et al. 2019; Felson and Ellison 2021). Additionally, by disproportionately affecting the predators that connect aquatic and terrestrial ecosystems (e.g., spiders, dragonflies), urbanisation may compartmentalise food webs, thereby weakening energy flows across ecosystems (Sullivan and Manning 2019). While local‐ and landscape‐scale improvements, such as increasing the quantity of urban green and blue spaces, are likely to increase species diversity—particularly among predators (Hironaka and Koike 2013; Egerer et al. 2017; Frey et al. 2018; Tobisch et al. 2023)—the resulting effects on food web dynamics remain unclear. By bolstering the diversity of urban predators, which are often generalists (Fournier et al. 2020), these improvements may lead to networks with longer chains, higher trophic levels, and omnivory (Table 1), which could reduce the risk of secondary extinctions (Table 1) (Kratina et al. 2012; Start et al. 2019). Such changes could further alter food web structure, resulting in more connected yet nested networks, as prey species become linked through shared predators, or to decreased interspecific diet niche overlap (henceforth: “niche overlap”), further stabilising food webs (Turnbull et al. 2013). Despite these possibilities, urban studies typically focus on simpler networks (e.g., plant‐pollinators; Casanelles‐Abella et al. 2022) or single ecosystems (e.g., terrestrial; Start et al. 2019), neglecting larger multitrophic dynamics across ecosystems.

**TABLE 1:** Definition of food web properties, as well as their potential ecological interpretation.

Properties	Type	Definition	Ecological interpretation	References
Node degree skewness	Composition	Skewness of the number of links entering and leaving nodes (i.e., degree)	A high node degree skewness indicates a disproportional representation of poorly (skewness > 0) or highly connected nodes (skewness < 0)	Dunne et al. (2002)
Mean trophic level	Composition	Mean number of links from basal resources to top predators	Longer food chains have higher mean trophic levels	Williams and Martinez (2004)
Generality	Composition	Mean number of preys per predator	Generality indicates higher levels of generalists	Schoener (1989)
Omnivory	Composition	Standard deviation of prey species' trophic level, averaged overall all consumers in the food web	Indicates the breadth of a consumers' diet and can be linked to stability	Williams and Martinez (2004)
Trophic incoherence	Composition	Degree of heterogeneity across differences in trophic levels between each pair of consumer‐resource	Trophic incoherence is linked to stability	Johnson et al. (2014)
Connectance	Structure	Proportion of realised interaction relative to all potential interactions across all nodes	Measures network complexity	Dunne et al. (2002)
Modularity	Structure	Tendency of networks to create subnetworks of interacting nodes	Could be linked to taxa using different sets of resources or becoming spatially isolated	Eskuche‐Keith et al. (2023)
Nestedness	Structure	The degree to which specialist taxa's diets are subsets of generalist taxa's diets, calculated using the paired overlap and decreasing fill metric	Nestedness is linked to higher food web stability	Thébault and Fontaine (2010)
Niche overlap	Structure	Jaccard similarity in the diet of nodes in a network	High niche overlap can indicate greater competition for food and may decrease stability	Turnbull et al. (2013), Ho et al. (2022)
Proportion of basal consumers	Composition	Proportion of taxa only feeding on basal resources. Includes primary consumers (nodes feeding on plants), but also other consumers of basal resources (e.g., detritivores)	Number of taxa feeding on basal resources (e.g., detritus, microbes, plankton) compared to total site richness	Galiana et al. (2021)
Proportion of predators	Composition	Proportion of taxa feeding at least on one other taxa.	Number of predators compared to total site richness.	This publication
Aquatic – terrestrial module relative size	Composition	Proportion of nodes clustered in the same modules as terrestrial or aquatic basal resources (i.e., aquatic and terrestrial detritus, respectively)	The more taxa are likely to be clustered with aquatic or terrestrial basal resources, the less likely they are to interact with other taxa in the food web	This publication

One way to study complex food webs is through a metaweb approach, which defines regional interaction pools and infers local food webs via species co‐occurrence (Dunne 2006). In urban environments, however, limited access to habitats (e.g., private ponds) and sparse trophic interaction data have historically hindered its application. Yet, recent advancements in environmental DNA (eDNA) metabarcoding, coupled with newly available species interaction databases (Reji Chacko et al. 2025), now enable comprehensive exploration of urban biodiversity and food web dynamics across ecosystem boundaries (Deiner et al. 2017; Hupało et al. 2022; Blackman et al. 2022), and are particularly well suited to addressing global biodiversity targets (Altermatt et al. 2025). In this study, coupling a metaweb approach with invertebrate‐specific eDNA metabarcoding data from 54 paired aquatic‐terrestrial sites in Zurich, Switzerland, we tested (1) whether urbanisation reduces predator prevalence, (2) whether this decline alters food‐web composition and structure, leading to more modular aquatic and terrestrial food webs, and (3) whether improvements in habitat quality and quantity at the local scale, along with connectivity and complementarity at the landscape scale, increase predator diversity and subsequently affect other food web properties (e.g., omnivory, nestedness).

## 2 Methods

### 2.1 Environmental DNA Collection and Processing

**FIGURE 1:**
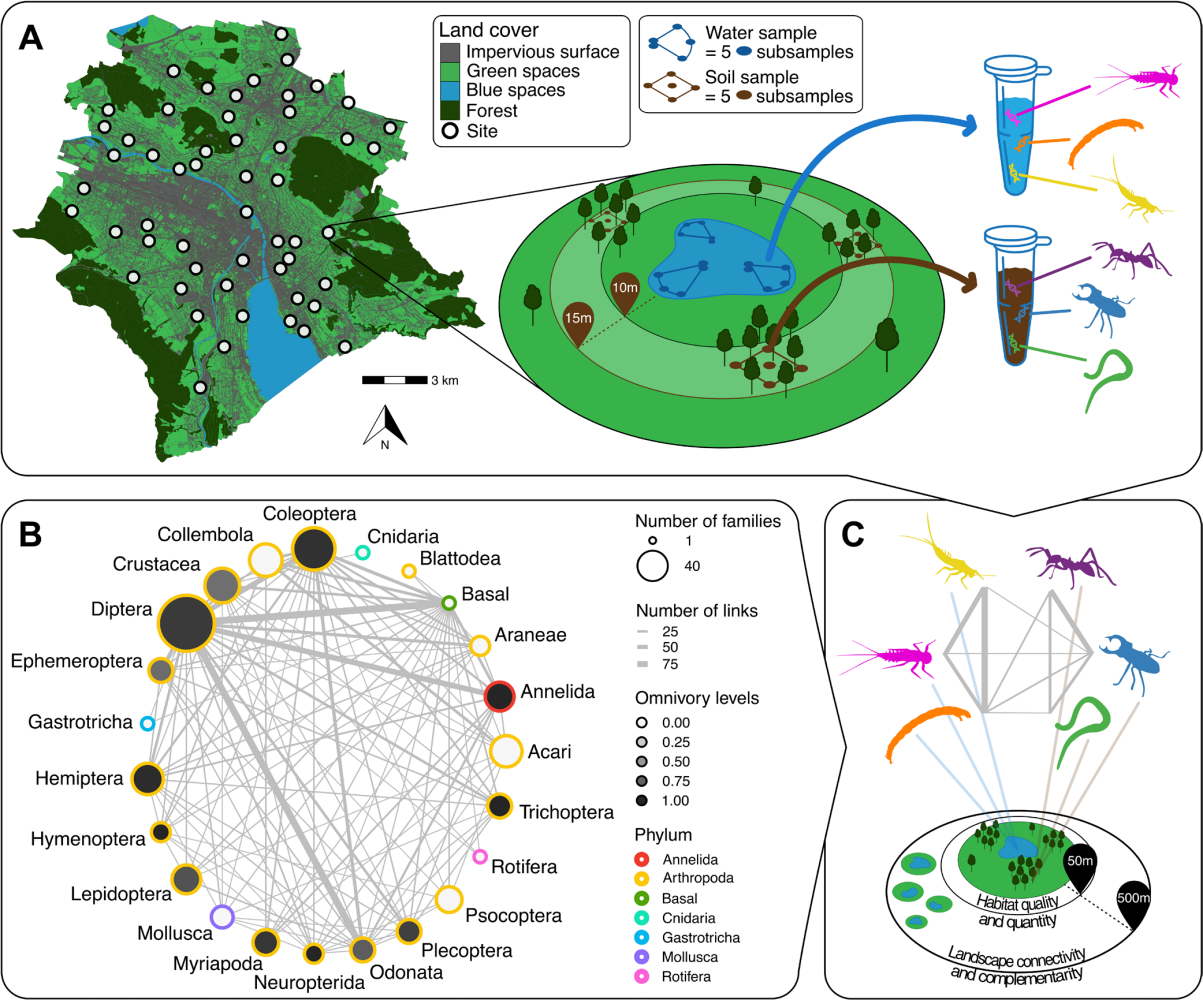
Environmental DNA sampling design and local food web inference. (A) Site location in the city of Zurich and sampling overview. Brown squares indicate soil sampling points 10–15 m from the pond in preserved vegetation patches. Blue triangles indicate water sampling points around the pond at two depth levels (under the surface and at the bottom of the pond, represented by overlapping dots). (B) Global metaweb for local metaweb inference (see C). Although resolved at the family level in all analyses, the metaweb is here depicted at phylum, class, or order levels for visualisation purposes. Circle size indicates the number of families, line thickness shows links between groups, filling colour represents the mean fraction of omnivorous families, and stroke colour denotes phylogenetic placement. Basal indicates basal resources (e.g., terrestrial detritus). (C) Example of combining species lists from (A) with the global metaweb (B) to infer a local food web and assess the effect of habitat quality and quantity (50 m buffer) and landscape connectivity and complementarity (500 m buffer).

Despite being densely built, the city of Zurich, Switzerland (92 km^2^, ~450,000 inhabitants), features 35 km^2^ of green spaces, 21 km^2^ of forest, and nearly 700 ponds (Figure 1A) (Stadt Zürich 2021). Given that urban growth is expected to occur primarily in small to mid‐sized cities—although often in economically weaker regions—Zurich, as a typical mid‐sized European city, serves as a valuable case study for urban growth (Seto et al. 2013). In 2022, water and soil eDNA samples (Pawlowski et al. 2020) were collected across 54 paired aquatic and terrestrial sites along an urbanisation gradient (Figure 1A), with urbanisation quantified as a composite proxy incorporating multiple land‐use, social, pollution, and climate metrics to capture the diverse characteristics of urban environments (Briski et al. 2025) (see *Environmental predictors*). While urbanisation can also encompass urban sprawl, we hereafter use the term urbanisation primarily to refer to urban densification.

All sites consisted of a pond enclosed within an urban green space (e.g., private garden, park) and were selected to capture a range of environmental conditions at the local (i.e., pond area, habitat patch size, and the proportion of vegetation within a 50‐m buffer) and landscape scale (proportion of sealed surfaces within a 500‐m buffer, distance from the forest, and distance to the city center). Site location was also considered to ensure good spatial coverage and minimal correlations between local and landscape factors (Spearman's rank correlation; max *R* ^2^ = 0.33).

At each site, three water and three soil samples were collected at different locations around the pond and the urban green space, respectively, to capture microhabitat diversity (Figure 1A). Each water sample involved filtering water using a Sterivex filter (0.22 μm pore size) until 1 L was filtered or until clogging, in which case a 100 μm pre‐filter was used to delay clogging. Daily negative field controls were included to check for contamination. Additionally, soil samples were collected under the least disturbed vegetation patches, from 10 to 15 m from the pond, by extracting and subsequently pooling samples from each corner and the center of 1 m^2^ quadrat using a soil core sampler (5 cm diameter, 5 cm depth). Both water and soil samples were stored at −20°C upon return to the lab, but soil samples were first sieved and homogenised using a 2 mm sieve.

Water and soil samples were then extracted in dedicated eDNA laboratories to prevent cross‐contamination using the Qiagen PowerWater Sterivex Extraction Kit and Qiagen PowerMax Soil Extraction Kit, respectively, along with frequent negative extraction controls. A two‐step PCR amplicon sequencing workflow was performed: each sample was amplified five times, targeting a fragment of the COI barcode using aquatic and terrestrial invertebrate‐specific primers (i.e., Terr_EPTDr2n primers, Perrelet et al. 2025). Amplified PCR replicates were then pooled, purified, and indexed. Following a second purification and quantification, samples were normalised, pooled to equimolar concentrations, and sequenced on an Illumina NovaSeq 6000. Details on the site selection, eDNA sampling, and sample processing are available in Perrelet et al. (2025).

### 2.2 Bioinformatics

We followed the same Perrelet et al. (2025) and assessed raw read quality, followed by primer removal, merging, quality filtering, dereplication, and OTU clustering. OTUs were filtered to remove sequences with abnormal lengths (±10 bp), high error rates (maxEE > 1), low read counts (< 10 reads), ambiguous bases (“N” s), or chimeras. Potential contaminations were addressed by subtracting the ratio of each OTU found in negative controls from those in the samples. Remaining OTUs were parsed using lulu v0.1.0 and rarefied to the 15th percentile to mitigate sequencing bias.

Representative sequences of all remaining OTUs were assigned to the MIDORI2 reference database using the IDTAXA algorithm in DECIPHER, with a 50% threshold for assignment retention. We excluded all unassigned OTUs, as well as the few that were assigned to non‐invertebrates. To align with the resolution of available trophic information, OTUs originating from the same families were aggregated. Given that water and soil samples provide complementary methods for biodiversity assessment (Perrelet et al. 2025), families identified independently in either water or soil samples at a given site were aggregated at the site level to represent family‐level biodiversity across both aquatic and terrestrial ecosystems at all 54 sites.

### 2.3 Metaweb and Local Food Web Inferences

To determine the composition and structure of local food webs across 54 sites, we used a metaweb approach, which involves aggregating all recorded interactions (in this present study: trophic relationships) among focal taxa (Figure 1B) in a pre‐defined region (Reji Chacko et al. 2025). We employed an existing Swiss national metaweb constructed from documented trophic interactions sourced from the literature, empirical natural history observations, and so‐far unpublished data from extensive field research (Reji Chacko et al. 2025). As some groups identified in the eDNA samples were not well represented by the original metaweb (e.g., soil fauna), additional trophic interactions were sourced from the literature in order to complete our metaweb (see Table S1).

For our analyses, the metaweb was resolved to the family level; thus, taxa within the same family were assumed to exhibit uniform feeding behaviours (e.g., all carabids were considered predators of springtails). This resolution was chosen as it is considered sufficient to monitor changes in trophic dynamics across environmental gradients (Thompson and Townsend 2000; Potapov et al. 2019), and allowed us to minimise biases towards well‐documented taxa while ensuring the inclusion of less‐documented groups, such as many soil fauna.

At each site, we inferred local food web interactions by combining our metaweb with a co‐occurrence‐based approach. We considered that if an interaction existed between two families in the global metaweb, it could potentially occur locally. Local food webs were thus subsets of the global metaweb, retaining only co‐occurring invertebrate families at each site. While co‐occurrence methods are increasingly used to address sampling challenges in biotic interactions (e.g., Blackman et al. 2022; Ho et al. 2022; Botella et al. 2024), their application to infer food webs across broad spatial and temporal scales has been criticised (Brimacombe et al. 2024). However, in this study, the limited transport and rapid decay of eDNA in topsoil (Valentin et al. 2021) and lentic water bodies (Troth et al. 2021) provide a reasonable rationale for assuming that co‐occurring and co‐interacting taxa are likely to engage in biotic interactions. Moreover, although urban environments can induce dietary shifts that our approach may not fully capture (Chen and Neoh 2023), this potential bias is likely consistent across sites. Moreover, since most diet shifts occur within the genus or family level (Pearse and Altermatt 2013), they would still be represented in our family‐level approach.

To capture variation in consumer trophic levels and address limited data on basal resource use, simplified basal resources, such as plants, fungi, plankton, and aquatic and terrestrial detritus, were considered present at each site, as these general categories are likely ubiquitous, and we assumed little variation in the availability of resources between sites. For each locally inferred food web (henceforth: local web), we quantified several common food web properties, including node degree skewness, mean trophic level, generality, omnivory, and trophic incoherence (compositional properties), as well as family richness, connectance, nestedness, niche overlap, and modularity (structural properties) (see Table 1). While partially correlated, these metrics were selected for their ability to offer a complementary view of network architecture changes linked to predator prevalence and identity (e.g., top, meso‐predator) (Figure S1). To assess modularity, we used a multi‐level community structure optimisation algorithm based on the Louvain method (Blondel et al. 2008). As the algorithm relies on random node ordering and local optimisation steps, it is non‐deterministic and a single measurement may not be representative. Therefore, we generated 100 community structures per local web and calculated the average modularity and number of modules. Additionally, we calculated the relative sizes of modules containing aquatic and terrestrial detritus, respectively, across iterations, as these modules likely included taxa associated with predominantly aquatic (e.g., *Daphnia*) or terrestrial (e.g., mites) ecosystems, with minimal interaction across ecosystem boundaries. To ensure that all above‐mentioned properties differed from random expectations, we created 100 randomised versions of each local web by shuffling trophic links while preserving node and link counts and keeping basal resources fixed. For each metric, we calculated its randomised counterpart as the mean across iterations.

Finally, we assessed potential changes in the proportion of predators and calculated the number of nodes with trophic levels > 2 relative to the expected overall trophic level across the metaweb (MacKay et al. 2020). Henceforth, consumers not classified as predators (i.e., those with trophic levels ≤ 2) are referred to as basal consumers.

### 2.4 Environmental Predictors

We quantified land use using a rasterized habitat map of Zurich at a 1‐m resolution (Stadt Zürich 2021), measuring five land‐use types across varying buffer sizes: (1) urbanisation and four proxies of biodiversity conservation strategies, specifically (2) habitat quality, (3) habitat quantity (both at 50 m buffer), (4) landscape connectivity, and (5) landscape complementarity (both at 500 m buffer).

1. *Urbanisation* was calculated as the first axis of a principal component analysis (PCA), collapsing several urban markers, including the fraction of impervious surfaces within a 500 m buffer (based on the habitat map; Stadt Zürich 2021), degree of overwarming (i.e., temperature deviation from the area‐wide average; Parlow et al. 2010), NO_2_ pollution (Stadt Zürich 2017), and human population density (Kanton Zürich 2020), following Casanelles‐Abella et al. (2021) and Dietzel et al. (2024) (Figure S2A).
2. *Habitat quality* was assessed using a similar PCA approach incorporating key drivers of both aquatic and terrestrial invertebrates: standard deviation of vegetation height, vegetation density, fraction of built‐up area, fraction of macrophytes within the pond, and proportion of concrete pond banks (Noordijk et al. 2010; Nielsen et al. 2014; Oertli and Parris 2019) (Figure S2A).
3. *Habitat quantity* was calculated based on the area covered by green spaces within a 50 m buffer around each pond margin, where increases in either green space cover or pond size would yield a larger habitat area.
4. *Landscape connectivity* was measured as the first axis of a PCA including the mean distance between ponds within a 500 m buffer, green patch cohesion index, distance to the nearest pond, and distance to the forest (Dietzel et al. 2024) (Figure S2A).
5. *Landscape complementarity* was assessed based on the number of distinct habitat types mapped in the habitat map (62 types total) in a 500 m buffer around ponds (Stadt Zürich 2021).

Moderate correlations (Pearson correlation coefficients: 0.50–0.61) were found between habitat quantity, landscape connectivity, and urbanisation; other variables were uncorrelated (Figure S2B).

### 2.5 Modelling

To evaluate the influence of the proportions of predators on food web properties, we used a series of (generalised) linear models. The response variables were various food web properties (following a normal distribution, except for species richness, which followed a Poisson distribution), while the proportion of predators served as predictive variables (normalised to meet model assumptions). We retrieved and standardised the results of each individual model, reporting the coefficients and 95% confidence intervals using coefficient plots.

To test direct and indirect effects of land‐use on interdependent food web properties, we conducted a series of piecewise structural equation modelling (SEM) analyses. SEM was chosen to disentangle the influence of environmental drivers from inherent correlations among food web metrics, some of which are mathematically related or highly collinear. We used a hierarchical modelling approach, first assessing land‐use effects on fundamental network properties (e.g., node degree skewness), then incorporating more complex emergent features (e.g., trophic incoherence). This structure ensured that effects on lower‐level properties were properly accounted for before evaluating their impacts on emergent properties. Expected relationships among food web properties are illustrated in Figure S3.

SEM relationships were derived from linear model analyses containing one land‐use type at a time as a fixed effect, as well as dependencies in either compositional or structural properties. The rationale was that if a driver altered predator‐to‐basal‐consumer ratios, urban predators, which are typically generalists (Fournier et al. 2020), would induce changes in the skewness of node degree distributions and mean trophic levels, which could subsequently affect generality, omnivory, and ultimately trophic incoherence (Johnson et al. 2014). The SEM analysis was extended to food web structural properties (see Table 1) using well‐established relationships from the literature (Ho et al. 2022). In short, if a driver (e.g., urbanisation) modulated the number of nodes, it could influence food web connectance (as mathematically determined by the number of nodes and links), in turn potentially facilitating the emergence of modules or nested structures within the food web, subsequently affecting the degree of niche overlap. Further details on SEM topologies are available in Data S1 and Figure S3.

To evaluate whether environmental drivers influenced food web properties directly or indirectly through intermediary food web properties, we calculated standardised path coefficients to distinguish direct effects (driver–response variable links) from indirect effects (mediated through other metrics), with significance assessed via bootstrapping (10,000 resamples, 95% CIs). We then repeated the same analysis, this time replacing the degree of urbanisation with the four above‐mentioned biodiversity conservation strategies, grouped based on spatial scale (i.e., local habitat quality and quantity or landscape connectivity and complementarity). Interactions between the two local and two landscape variables respectively were considered, but as they did not produce major changes in the results, only results from additive models are displayed here.

All analyses were conducted using R v4.4.1 (R Core Team 2024). The following packages were used in the analysis: *landscapemetrics* v2.1.1, *exactextractr* v0.10.0, *lidaRtRee* v4.0.5 (covariates calculations; Hesselbarth et al. 2019; Baston and ISciences 2023; Monnet 2023); *piecewiseSEM* v2.3.0.1, *semEff* v0.7.2 (modelling; Lefcheck et al. 2024; Murphy 2024).

## 3 Results

**FIGURE 2:**
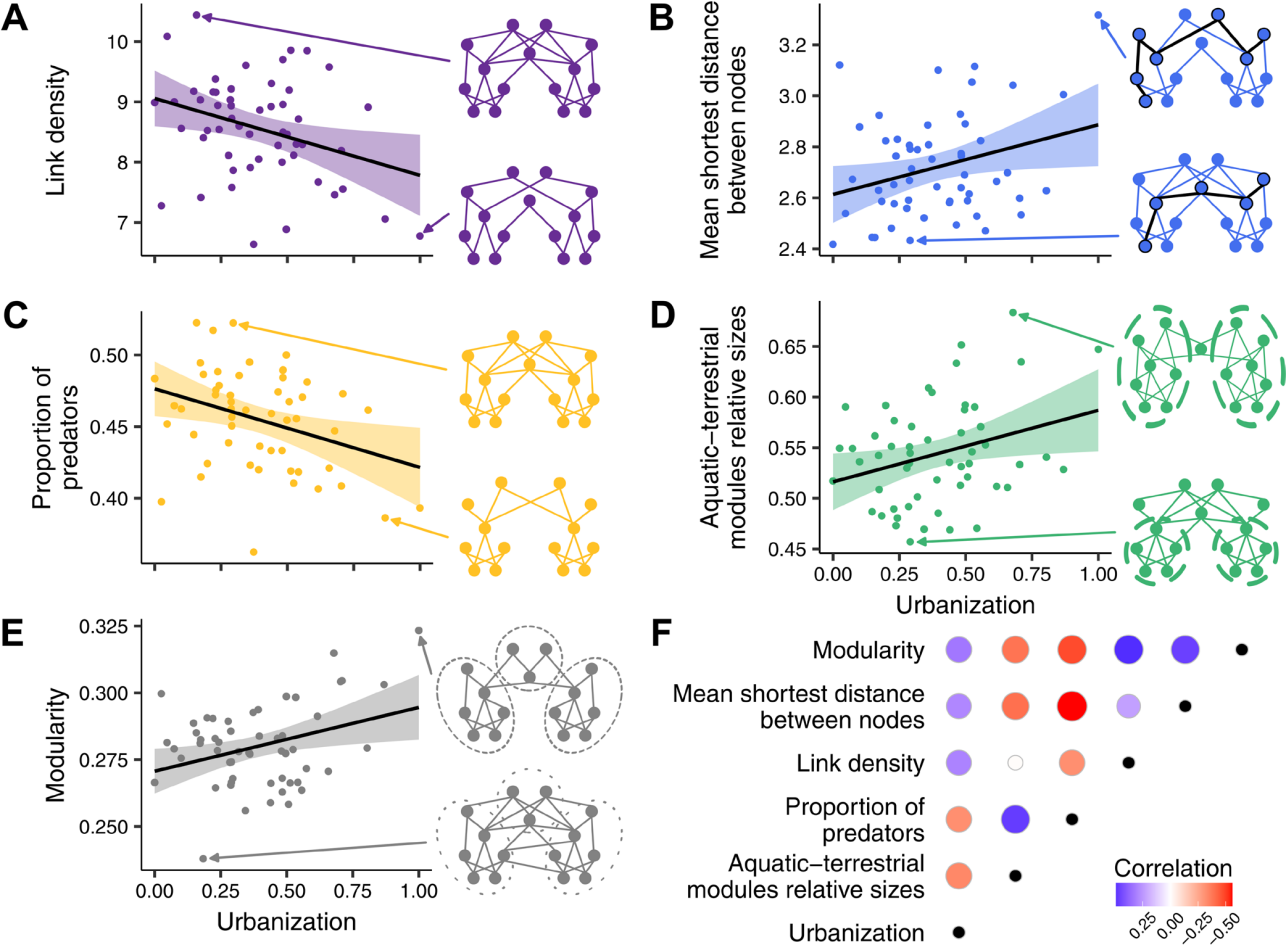
The influence of urbanisation on various food web properties: (A) link density, (B) distance between nodes, (C) proportion of predators, (D) relative module sizes for aquatic and terrestrial components, (E) modularity, and (F) correlation matrix of these variables. In (F), self‐ and non‐significant correlations are denoted by a black and white dot, respectively. Diagrams illustrating expected changes in food web structure and composition are provided for each property. All correlations shown here are significant. Hypothesized causal relationships between these variables are detailed in Figure S4.

Urbanisation significantly influenced the composition and, ultimately, structure of local food webs, leading to simpler (i.e., reduced link density) and more loosely connected (i.e., increased mean shortest path between nodes) networks (Figure 2A,B). Although family richness (i.e., number of nodes) was not significantly affected, urbanisation drove a trophic shift from predators to basal consumers and increased the relative size of aquatic and terrestrial modules (Figure 2C,D). The number of modules, however, remained stable (Figure S4). Predator loss and increasing compartmentalisation into aquatic and terrestrial modules jointly led to an indirect but positive correlation between urbanisation and modularity (Figure 2E and Figures S5 and S6). All food web properties differed from null model expectations (Figure S7), and aside from predator prevalence with module size and mean shortest distance between nodes, all properties were significantly interrelated (Figure 2F).

**FIGURE 3:**
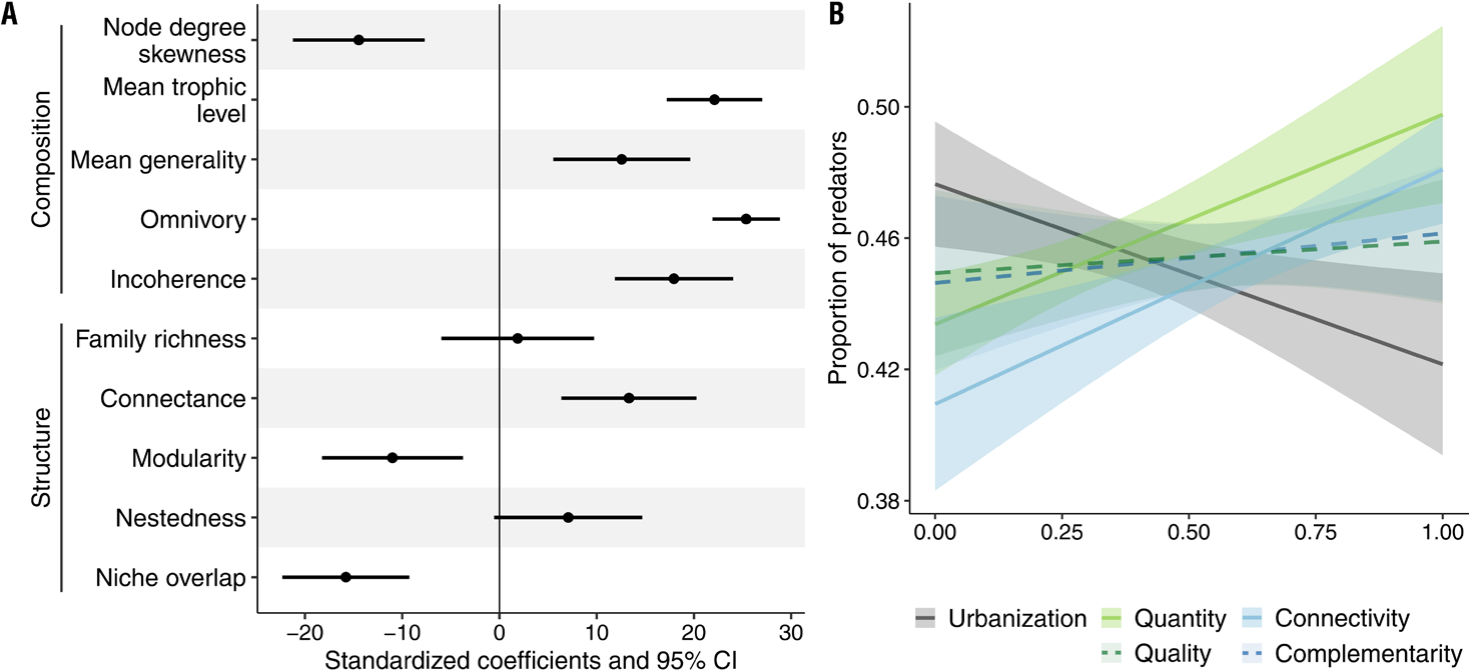
The proportion of predators based on habitat composition and configuration and its correlations with food web structural and compositional properties. (A) Coefficient plot showing the standardised coefficients and 95% confidence intervals of multiple independent linear models based on the proportion of predators on various food web structural and compositional properties (y‐axis), including: Skewness of node degree distribution, mean trophic level, generality, omnivory, and trophic incoherence (compositional), and family richness, connectance, modularity, nestedness, and niche overlap (structural). (B) Regression lines of the proportion of predators with urbanisation as well as all biodiversity conservation strategies (scaled 0–1). Non‐significant correlations are marked by a dashed line and lighter colour shades.

The proportion of predators emerged as central in shaping both the composition and structure of food webs (Figure 3A). Their presence correlated with increased predator‐to‐prey ratios (i.e., higher generality) and more mixed trophic‐level diets (i.e., omnivory), which contributed to networks exhibiting higher trophic incoherence. By linking with other predators and basal consumers, predator prevalence favoured more connected and nested food webs, while preventing modularity and niche overlap that would otherwise arise from basal consumers being linked to the same few resources (e.g., plants, aquatic detritus, fungi) (Figure 3A). However, while basal consumer richness remained stable, we observed a decrease in predator richness with increasing urbanisation (Figure S8), leading to a sharp decline in the proportion of predators relative to basal consumers (Figure 3B). Habitat quantity and landscape connectivity had the opposite effect as urbanisation, although habitat quantity consistently had the highest proportion of predators (Figure 3B and Figure S8). Interestingly, habitat quantity and landscape complementarity had no significant effect on predator proportions nor richness.

**FIGURE 4:**
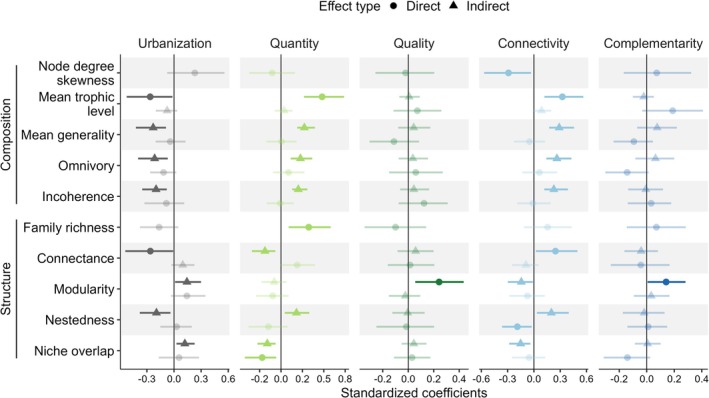
Direct and indirect effects of urbanisation and biodiversity conservation strategies on food web composition and structure. Shapes indicate effect types (round for direct, triangle for indirect), with lines representing 95% confidence intervals derived from bootstrapped standardised coefficients (10,000 resamples). Non‐significant relationships appear in lighter shades. The underlying structural equation models from which these effects are derived are presented in Figure S9.

Urbanisation triggered changes in food web composition and structure. In particular, SEM results highlighted that urbanisation had a marginally positive association with skewness in node degree distribution and a significant negative effect on mean trophic levels (Figure 4 and Figure S9), ultimately leading to higher prey‐to‐predator ratios (increased generality), reduced omnivory, and decreased trophic incoherence (Figure 4). In terms of network structure, although it did not impact family richness, urbanisation promoted lower network connectance (Figure S9), yielding less nested, more modular food webs, with greater niche overlap (Figure 4).

Improvements to local habitat quantity and quality, landscape connectivity and complementarity tended to yield effects that were opposite to those of urbanisation (Figure 4). Specifically, habitat quantity and landscape connectivity emerged as the most influential biodiversity conservation strategies, significantly impacting both food web composition and structure. Landscape connectivity reduced node degree distribution skewness, and, together with habitat quantity, increased mean trophic levels, indirectly enhancing generality, omnivory, and trophic incoherence (Figure 4 and Figure S9). However, neither habitat quality nor landscape complementarity showed a correlation with any compositional properties. Regarding food web structure, habitat quantity and landscape connectivity affected family richness and connectance, respectively (Figure 4 and Figure S9), with landscape connectivity indirectly negatively affecting modularity, and both landscape connectivity and habitat quantity indirectly positively associated with nestedness (Figure 4). Furthermore, although landscape connectivity was also indirectly associated with niche overlap, habitat quantity had a further direct impact of reducing niche overlap, beyond expectations based solely on species richness (Figure 4). Finally, the SEM analysis also showed that although habitat quality and landscape complementarity did not affect family richness or connectance, both factors directly contributed to increased modularity within food webs. For all SEM analyses, model fit was adequate (non‐significant Fisher's C). Further details on model fit and coefficients are available in Tables S2–S4.

## 4 Discussion

We found that urbanisation significantly decreased the prevalence of predators, thereby altering both the composition and structure of local food webs and weakening the links between aquatic and terrestrial ecosystems, consistent with our expectations. Our findings also support the idea that local and landscape‐scale biodiversity conservation strategies can influence food web composition, particularly at higher trophic levels, resulting in more complex, vertically diverse, and ultimately stable food webs.

Urbanisation exerted a multifaceted, often indirect, impact on food webs, creating simpler (i.e., lower link density) and more loosely connected networks (i.e., greater node distance). Although reduced link density could suggest increased specialist diversity (Tylianakis and Morris 2017), we attribute it to the replacement of highly connected predators with basal consumers linked to a few broadly defined resources (e.g., aquatic detritus, fungi, plants). Given the generalist nature of urban predators, this shift likely reflects predator‐specific vulnerabilities to urban stressors (e.g., Egerer et al. 2017) or potential bottom‐up effects (e.g., bioaccumulation, shift in prey composition; Jones and Clark 1987), and may indicate a loss of top‐down regulation enabling basal consumer proliferation (i.e., enemy release hypothesis; Crooks and Soulé 1999). Declining predator diversity also skewed node degree distributions and reduced connectance. Consequently, while urbanisation may increase connectance in specialised pest‐parasitoid networks due to a growing prevalence of generalists (Start et al. 2019), our results suggest the opposite in broader invertebrate consumer networks, leading to simpler, less connected, and more homogeneous food webs, compromising their stability (Dunne et al. 2002). Furthermore, although dependent on interaction strength (Gellner and McCann 2012), the negative indirect effect of urbanisation on omnivory may reduce food webs' ability to buffer environmental change (Kratina et al. 2012), further reinforcing urbanisation's destabilising influence (Felson and Ellison 2021). Predator loss may also undermine top‐down control crucial for ecosystem functions, such as pest control and pollination (Knight et al. 2005; Wang and Brose 2018).

Aquatic‐terrestrial food webs reflect a shared evolutionary and ecological history (Sullivan and Manning 2019; Twining et al. 2021; McFadden et al. 2022), yet in novel and relatively recent urban environments (Aronson et al. 2016), increasing urbanisation led to food web compartmentalisation into distinct aquatic and terrestrial modules. Predator decline and the simultaneous growth of aquatic and terrestrial modules reduced cross‐ecosystem links and energy transfers between aquatic and terrestrial ecosystems (Sullivan and Manning 2019). Consequently, modularity increased due to a rise in isolated basal nodes and fewer connecting predators. While modularity may limit the spread of disturbances (Thébault and Fontaine 2010), it may here indicate a weakening of the links between aquatic and terrestrial food webs. Although aquatic‐terrestrial coupling may still occur through other pathways (e.g., leaf litter, floods; Schulz et al. 2015; Gounand et al. 2018), these results highlight the role of predators in connecting ecosystems (Sullivan and Manning 2019).

Despite the destabilising influence of urbanisation, improvements at both local and landscape scales (i.e., bettering habitat quantity, quality, connectivity, and complementarity) generally promoted food web complexity. Among conservation strategies, habitat quantity and connectivity had the strongest effects on food web properties, both directly and indirectly. In contrast, habitat quality and landscape complementarity had more limited impacts, likely due to challenges in capturing community‐wide proxies for these factors, which vary with species‐specific needs (Oertli and Parris 2019). While vegetation structure is often highlighted as a key driver of aquatic (Oertli and Parris 2019) and terrestrial (Nielsen et al. 2014) communities, our proxies may not fully reflect all species' preferences. Nevertheless, the observed positive impact of habitat quality and complementarity on modularity suggests potential benefits for specialist species and enhanced food web stability (Thébault and Fontaine 2010; Johnson et al. 2014).

Increasing habitat quantity was particularly effective at enhancing predator prevalence, likely due to the sensitivity of predators to local land‐use change factors (e.g., vegetation cover) (Newbold et al. 2020; Tobisch et al. 2023). By promoting more trophically diverse systems, habitat quantity was associated with higher trophic levels, increased generality, and greater omnivory. Additionally, habitat quantity was associated with increased family richness, indirectly leading to more nested networks, which may increase network resilience by enabling multiple pathways of energy flow and thus decrease the risks of secondary extinction (Dunne et al. 2002; Thébault and Fontaine 2010). Additionally, by supporting assemblages spanning trophic levels, habitat quantity decreased the proportion of nodes connected to few broad basal resources (e.g., plants), thereby decreasing niche overlap and thus potentially reducing competitive exclusion risk and increasing stability (Turnbull et al. 2013). However, this shift towards larger, more complex networks coincided with increased trophic incoherence, which could undermine stability by amplifying the risk of destabilising feedback loops and unpredictable interactions (Johnson et al. 2014; Johnson and Jones 2017), although the extent of this effect will depend on interaction strength (O'Gorman et al. 2011).

Landscape connectivity, while not directly affecting family richness, promoted the inclusion of predators in food webs, leading to a less skewed distribution of node degree and higher trophic levels. These changes led to more connected, nested, and less modular networks, which may favour network stability and resilience through more flexible energy flows (Thébault and Fontaine 2010). However, landscape connectivity had a smaller impact than habitat quantity on reducing niche overlap, likely due to the high dependence of predators on local factors (e.g., spiders) (Egerer et al. 2017), leading to networks containing a higher proportion of basal consumers, even at the highest levels of landscape connectivity. This suggests that while landscape connectivity can improve food web complexity, its stabilising effects may be limited without concurrent improvements in habitat quality that support predators (Egerer et al. 2017; Tobisch et al. 2023).

Overall, this study underscores the complex interplay between urbanisation, local or landscape‐scale habitat improvements, and food web structure. Future studies should explore how colonisation or extinction of other taxa, even within a single realm (e.g., birds, fish), might reshape aquatic‐terrestrial food webs—though differences in dispersal abilities (e.g., birds vs. taxa studied here) will represent a challenge. Moreover, a deeper understanding of basal resource use could help clarify how urbanisation affects specialists at lower trophic levels. While plant barcode amplification could have improved resource characterisation, wind‐borne pollen dispersal may have led to resource diversity overestimation (Johnson and Barnes 2024), particularly given the small spatial scale of Zurich. Site‐level plant surveys represented a suitable alternative but were impractical due to the large number of sites, their inaccessibility due to private ownership, and extensive taxonomic knowledge of native and non‐native plants (both aquatic and terrestrial). Sampling private property also limited our ability to assess deviations from known feeding behaviours in the metaweb. Finally, while eDNA metabarcoding was key in addressing challenges associated with sampling private property, it often cannot yield quantitative results (Blackman et al. 2024). In order to derive weighted food webs, future studies should quantify species biomass and interaction strength, as these factors will impact energy fluxes between aquatic and terrestrial ecosystems (Shipley et al. 2024). Our approach, while limited in this regard, still effectively captured multitrophic dynamics at fine taxonomic resolution across an urbanisation gradient. Ultimately, our findings emphasise that although urbanisation simplifies food webs, enhancing habitat quantity and landscape connectivity can help mitigate these effects by promoting complexity and stability.

## 5 Conclusion

Urbanisation leads to the loss, simplification, and fragmentation of blue and green habitats, threatening aquatic and terrestrial invertebrate communities alike. Our results show that the effects of urbanisation extend beyond the direct impacts on individual ecosystems, significantly influencing the structure of interconnected aquatic and terrestrial food webs. In Zurich, Switzerland, we found that urbanisation (specifically, urban densification) leads to the simplification, homogenisation, and decoupling of aquatic and terrestrial food webs, as predators—forming nodes connecting both realms—are filtered out. The loss of these nodes not only diminishes overall biodiversity but may also destabilise urban aquatic and terrestrial ecosystems, potentially increasing their vulnerability to disturbances. However, our findings suggest that enhancing habitat quality and improving landscape connectivity can mitigate some of these effects, fostering more complex, nested, and interconnected aquatic and terrestrial food webs. In particular, thoughtful local and landscape‐scale biodiversity conservation strategies for biodiversity conservation might offer a way to restore predator communities, which play a pivotal role in maintaining food web structure and stability.

## Author Contributions

K.P., L.M.C., F.A., and M.M. conceived and designed the study. K.P. and M.R.C. collected the data. K.P. analysed the data. K.P. wrote the initial draft. All authors commented on and approved the final manuscript.

## Conflicts of Interest

The authors declare no conflicts of interest.

## Peer Review

The peer review history for this article is available at https://www.webofscience.com/api/gateway/wos/peer‐review/10.1111/ele.70212.

## Supporting information


**Data S1:** ele70212‐sup‐0001‐DataS1.docx.

## Data Availability

Sequencing data generated during this study is available on the European Nucleotide Archive under accession project numbers PRJEB88517. Analysis files and R script are available on GitHub (https://github.com/KPerrelet/Urban_food_webs) and Zenodo (https://doi.org/10.5281/zenodo.16947003).
