# Supplementary material for: Urbanisation Drives the Decoupling, Simplification, and Homogenization of Aquatic and Terrestrial Food Webs
Source: Ecol Lett. 2025 Sep 14;28(9):e70212. doi: 10.1111/ele.70212 (PMC12433592; doi:10.1111/ele.70212)
Supplement: Supplementary file 1 — Data S1: ele70212‐sup‐0001‐DataS1.docx. [file ELE-28-0-s001.docx]

**Supplementary Materials for Urbanization drives the decoupling, simplification, and homogenization of aquatic and terrestrial food webs**

*Supplementary Materials I*

Building on the complex interdependencies among food web compositional and structural properties, we developed a piecewise structural equation modeling (SEM) framework to capture both direct land-use effects and cascading indirect effects mediated by interconnected food web variables. Since properties such as node degree skewness, mean trophic level, omnivory, generality, and trophic incoherence are correlated and often mathematically derived from each other—with lower-level changes influencing higher-order traits—our SEM explicitly represents these causal pathways. Figure S3 presents a summary of expected relationships between food web properties.

We adopted a hierarchical modeling approach, first assessing the effects of land use on fundamental, low-level network metrics (e.g., node degree distribution), before expanding to broader food-web properties (e.g., omnivory, trophic incoherence). Given that the food webs studied are highly generalist—with predators linked to many nodes and feeding across multiple trophic levels (see Fournier et al., 2020)—there is a strong correlation between node degree and trophic level. Predators also prey on basal consumers and on one another, linking predator diversity to prey number. Accordingly, we first modeled land-use effects on node degree skewness, then on mean trophic level, including node degree skewness as a covariate to control for their correlation (see Fig. S3). Generality was modeled as a function of node degree skewness (reflecting link distribution used to mathematically calculate generality) and mean trophic level (accounting for potential increases in prey diversity due to predator-predator interactions). Omnivory, influenced by the generalist feeding behavior of predators across trophic levels, was also modeled based on node degree skewness and mean trophic level. Due to the strong association between omnivory and trophic incoherence (Johnson et al., 2014), we included omnivory to the lower-level metrics when modeling incoherence. Land use was included in all models to account for direct effects beyond those mediated by the lower-level variables.

Structural properties were modeled following a similar rationale, grounded in established mathematical relationships and prior literature (see Ho et al., 2022). Taxonomic richness (number of nodes) and connectance (proportion of realized links) are mathematically linked, so taxonomic richness was included when modeling connectance to account for land-use effects (see Fig. S3). Connectance and richness jointly influence network structure: highly connected networks tend to be less modular, while poorly connected networks rarely exhibit high nestedness, especially with generalist predators. Therefore, we included taxonomic richness and connectance as predictors when modeling nestedness and modularity, respectively. These metrics jointly inform the arrangement of trophic relationships, a key driver of species coexistence and niche partitioning (Ho et al., 2022; Turnbull et al., 2013). Consequently, niche overlap was modeled as a function of nestedness, modularity, and lower-level properties including taxonomic richness and connectance. As with compositional properties, land-use was incorporated in all models to capture direct effects not explained by changes in lower-level variables.

Full references are available at the end of this document


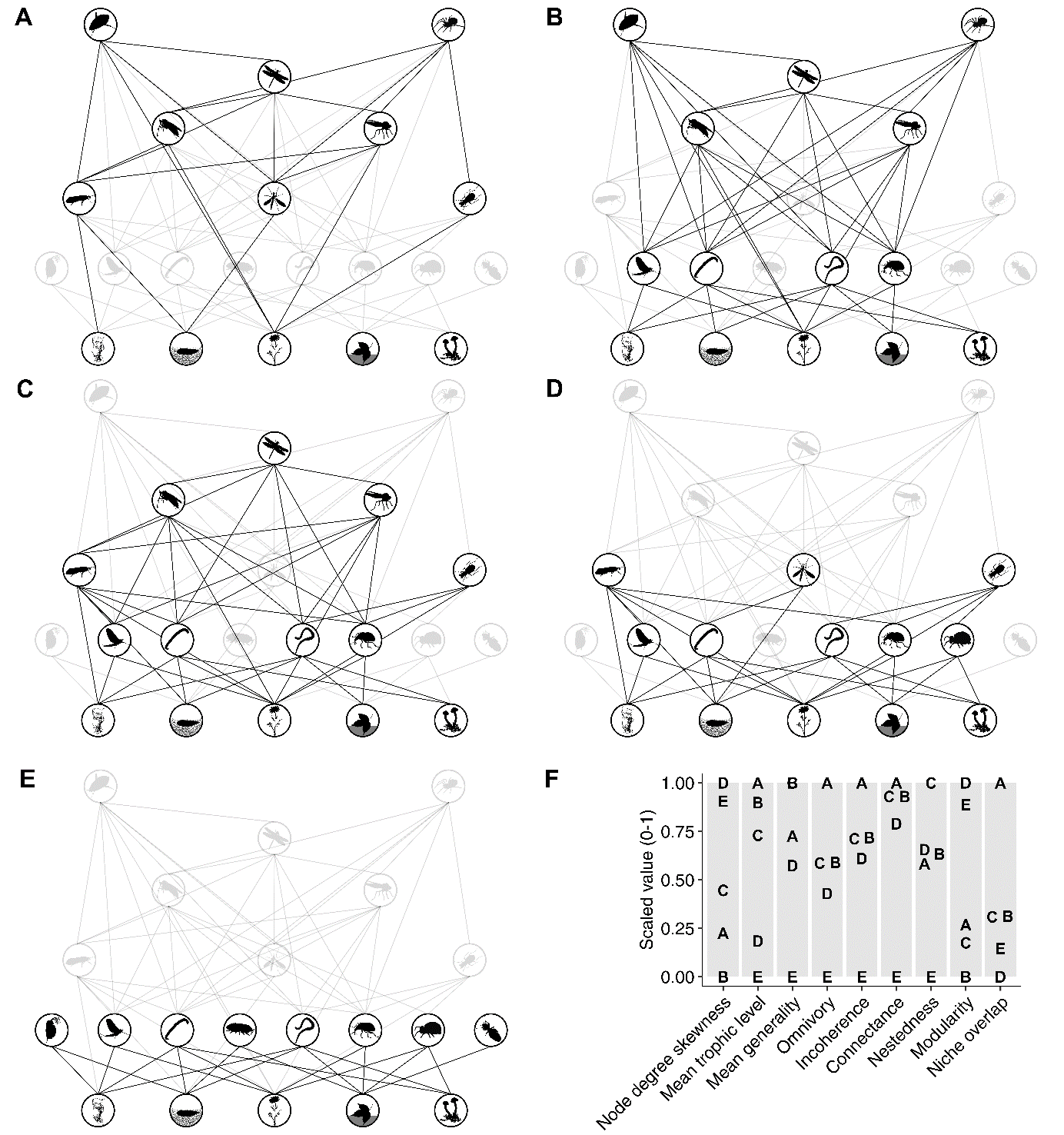
Figure S1: Schematic illustration of food webs differing in predator diversity and identity, and their resulting effects on various food web properties. All webs contain the same number of taxa (n = 8) and include resource nodes. Configurations include: (A) only predators, (B-C) combinations of low and high trophic-level predators with basal consumers—where high (B) or low (C) trophic-level predators dominate, (D) low trophic-level predators and basal consumers, and (E) only basal consumers. (F) Associated food web properties, including node degree skewness, mean trophic level, generality, omnivory, incoherence (compositional), and connectance, nestedness, modularity, and niche overlap (structural), exhibit substantial variation (scaled 0-1). Links and metrics reflect those from the regional metaweb (Reji Chacko et al., 2024; full references at the end of this document). Node placement mainly reflects both trophic level, but were moved to some extent for visualized purposes and avoid link overlaps.


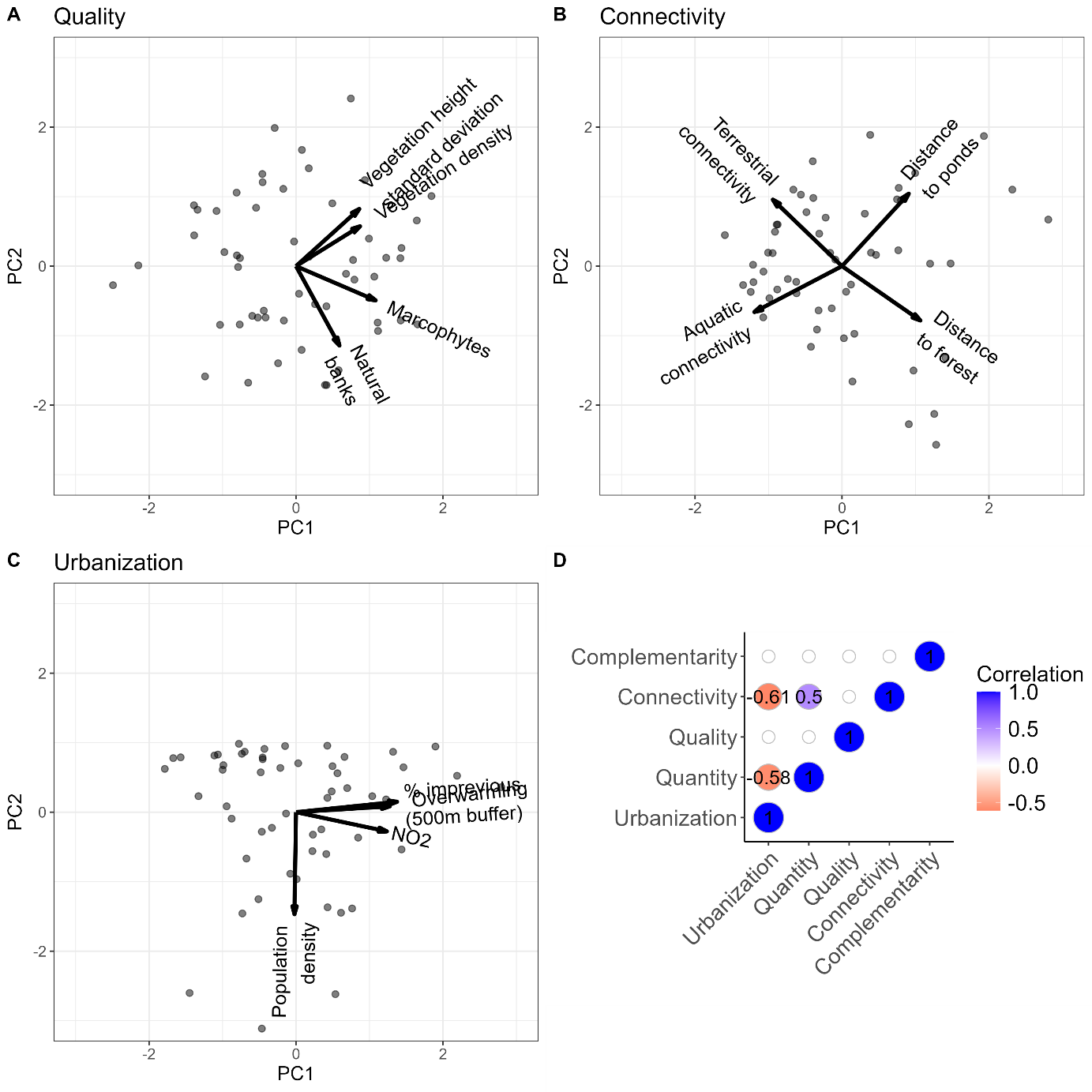
Figure S2: PCAs and correlation of environmental variables. (A) PCA biplot relationships among candidate variables. The first axis of each PCA was used to represent the composite variables. (B) Correlation matrix between the environmental variables. White dots indicate non-significant correlations, others are highly significant (p < 0.0001) and color-coded by strength.


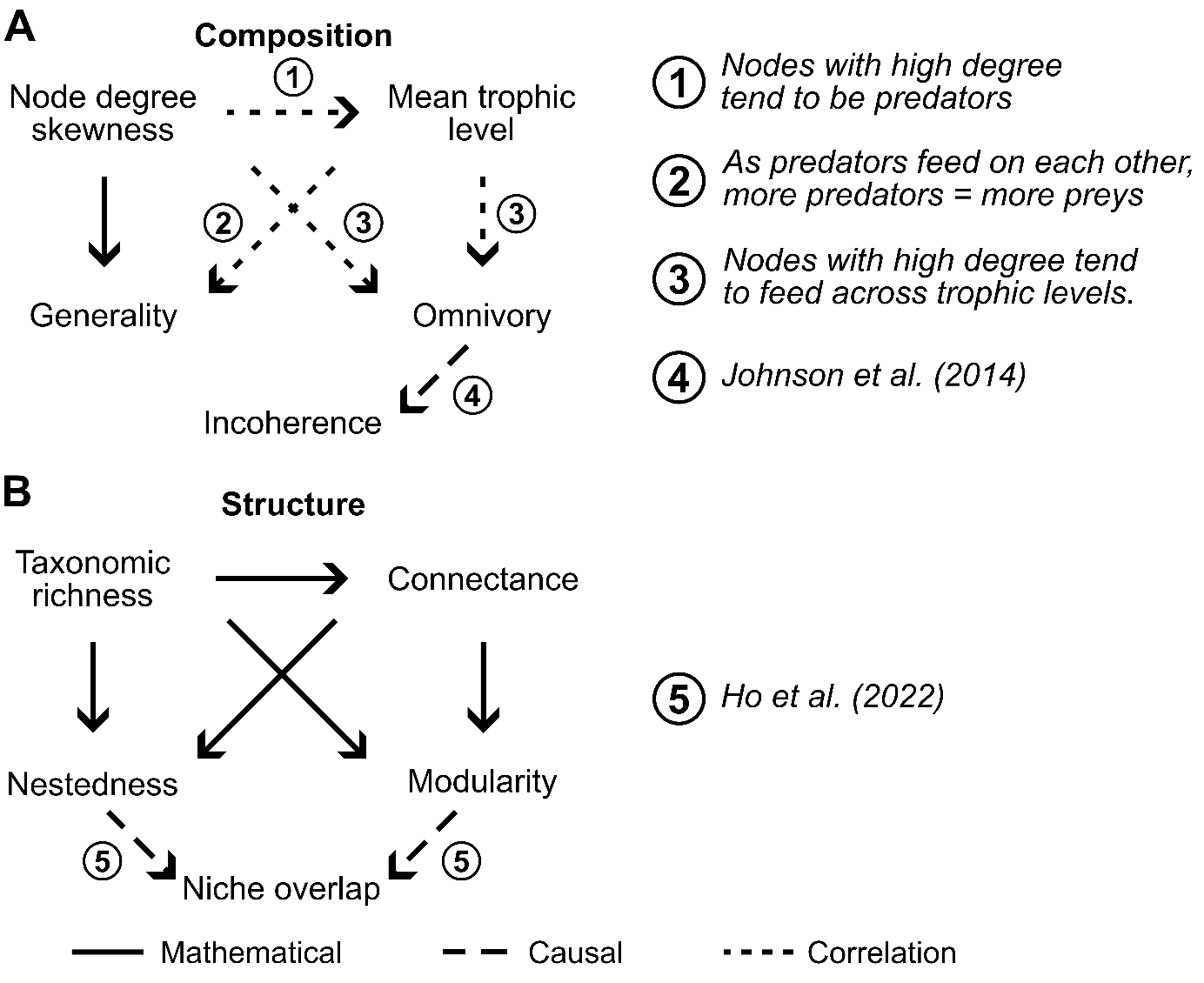
Figure S3: Conceptual diagram of the hypothesized relationships between food web properties included in the structural equation models (SEM). Arrows indicate expected directions of influence, with line types distinguishing relationship types: mathematical (one property defines another), causal (one property directly affects another without being mathematically derived), and correlational (properties tend to co-vary). In correlational cases, simpler food web properties are expected to influence more complex, emergent properties. Justifications for each link are provided alongside the diagram. Detailed SEM topologies are available in Supplementary Materials I. Full references are available at the end of this document.
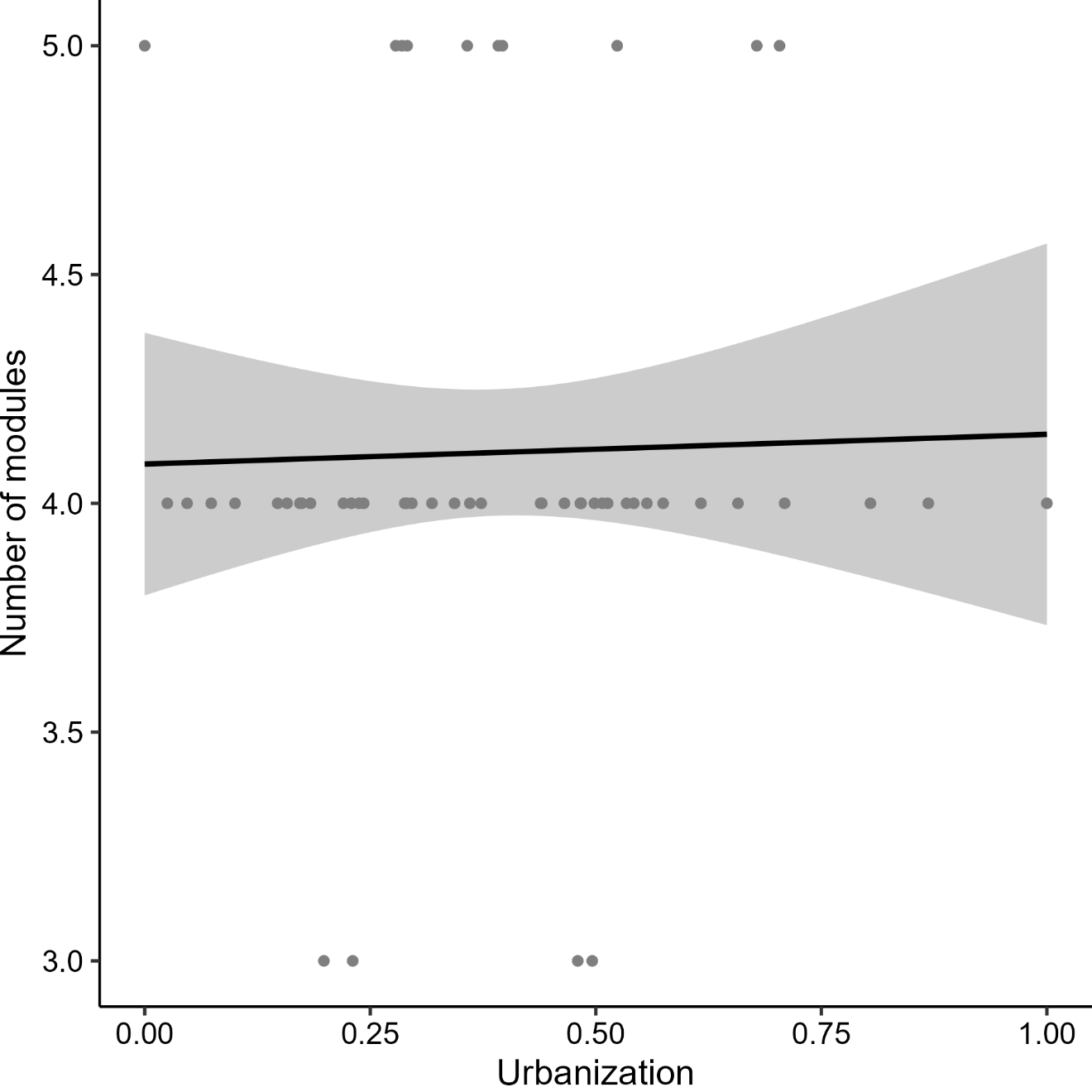
Figure S4: Regression lines of the number of modules with urbanization (scaled 0-1). The relationship is non-significant.


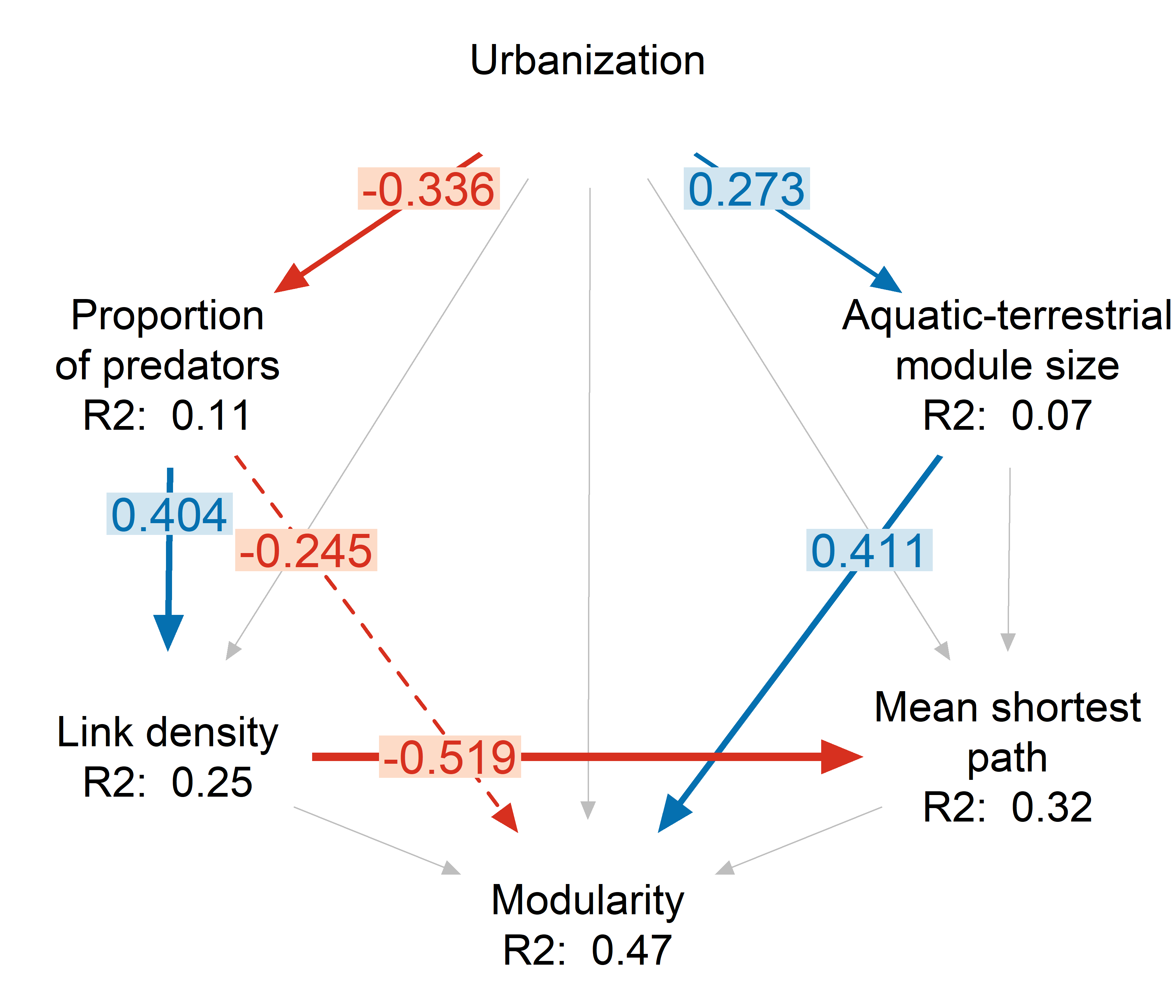
Figure S5: Structural equation model of the effects of urbanization on modularity. Blue arrows represent significant positive relationships, while red arrows indicate significant negative relationships (alpha ≤ 0.05). Arrow thickness corresponds to the coefficient size. Grey arrows denote non-significant relationships, with their coefficients not reported.


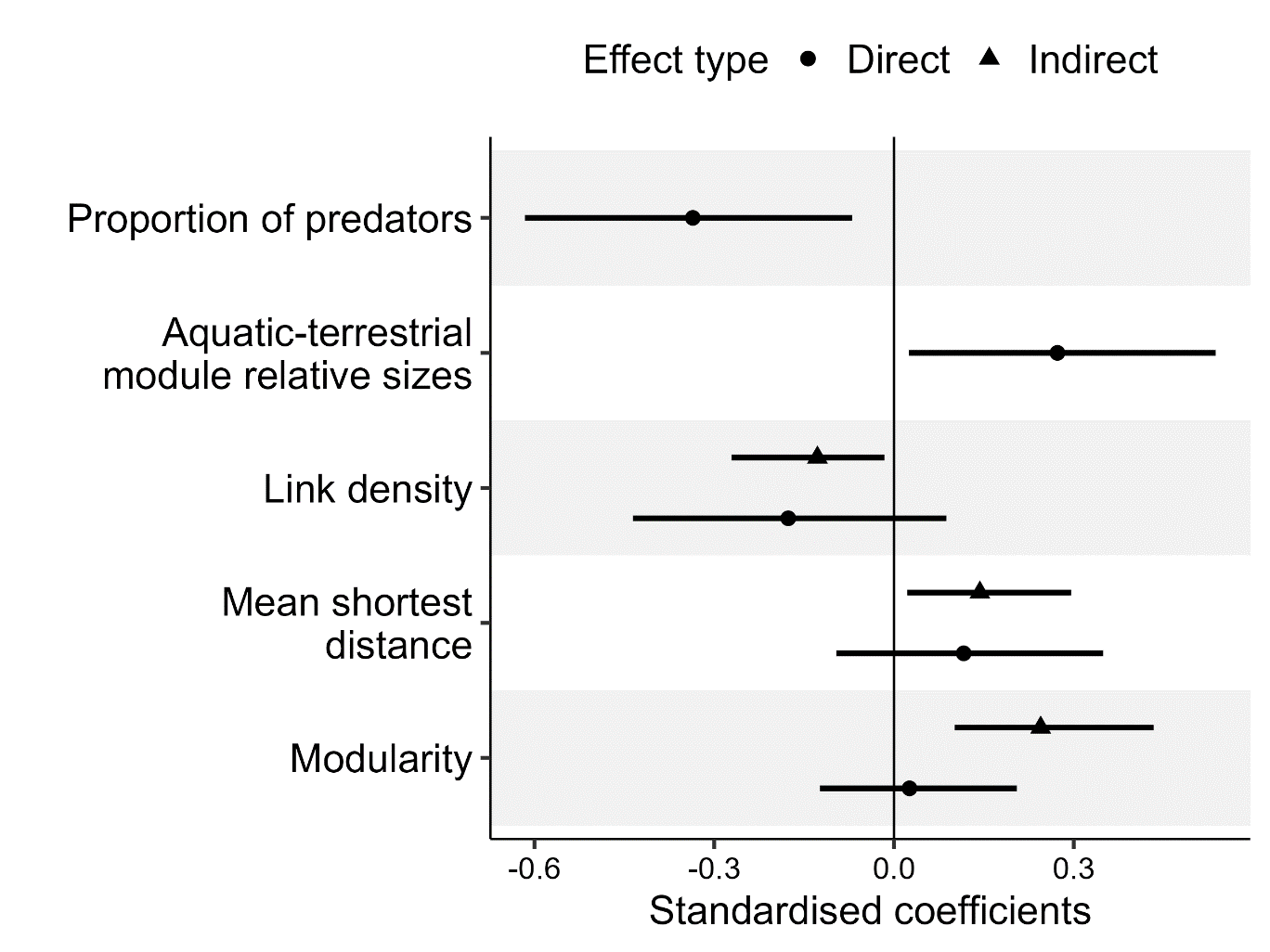
Figure S6: Direct and indirect coefficients between urbanization and food web properties pertaining from a structural equation model (see Fig. S5), based on bootstrapping of the standardized coefficients (10,000 replacements). Shape indicated direct (round) and indirect (triangle) effects, with lines indicating 95% confidence interval.
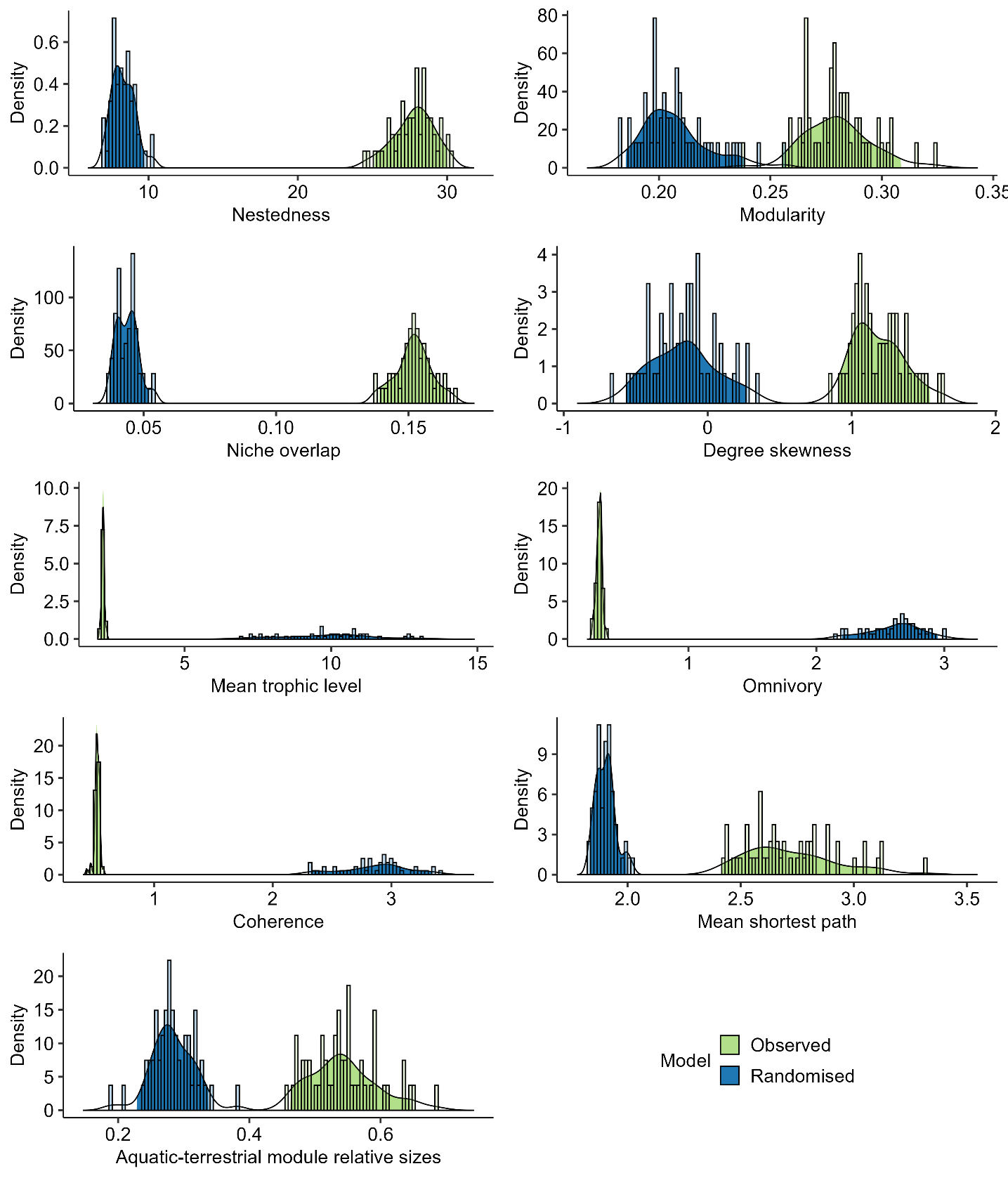
Figure S7: Null models of various food web properties. Observed properties are depicted in green, and properties of random food web are depicted in blue.


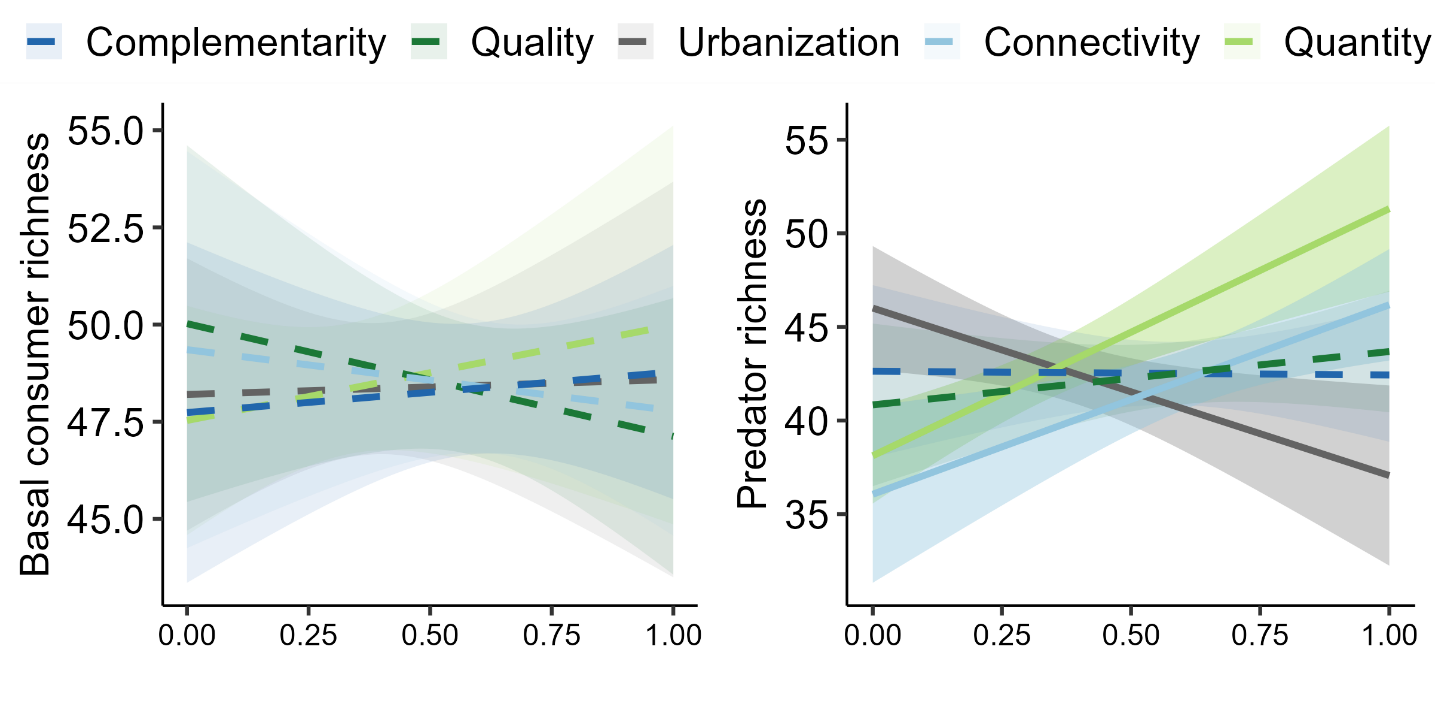
Figure S8: Regression lines of basal consumer (left) and predator (right) richness with urbanization as well as all biodiversity conservation strategies (scaled 0-1). Non-significant correlations are marked by a dashed line and lighter color shades.

*
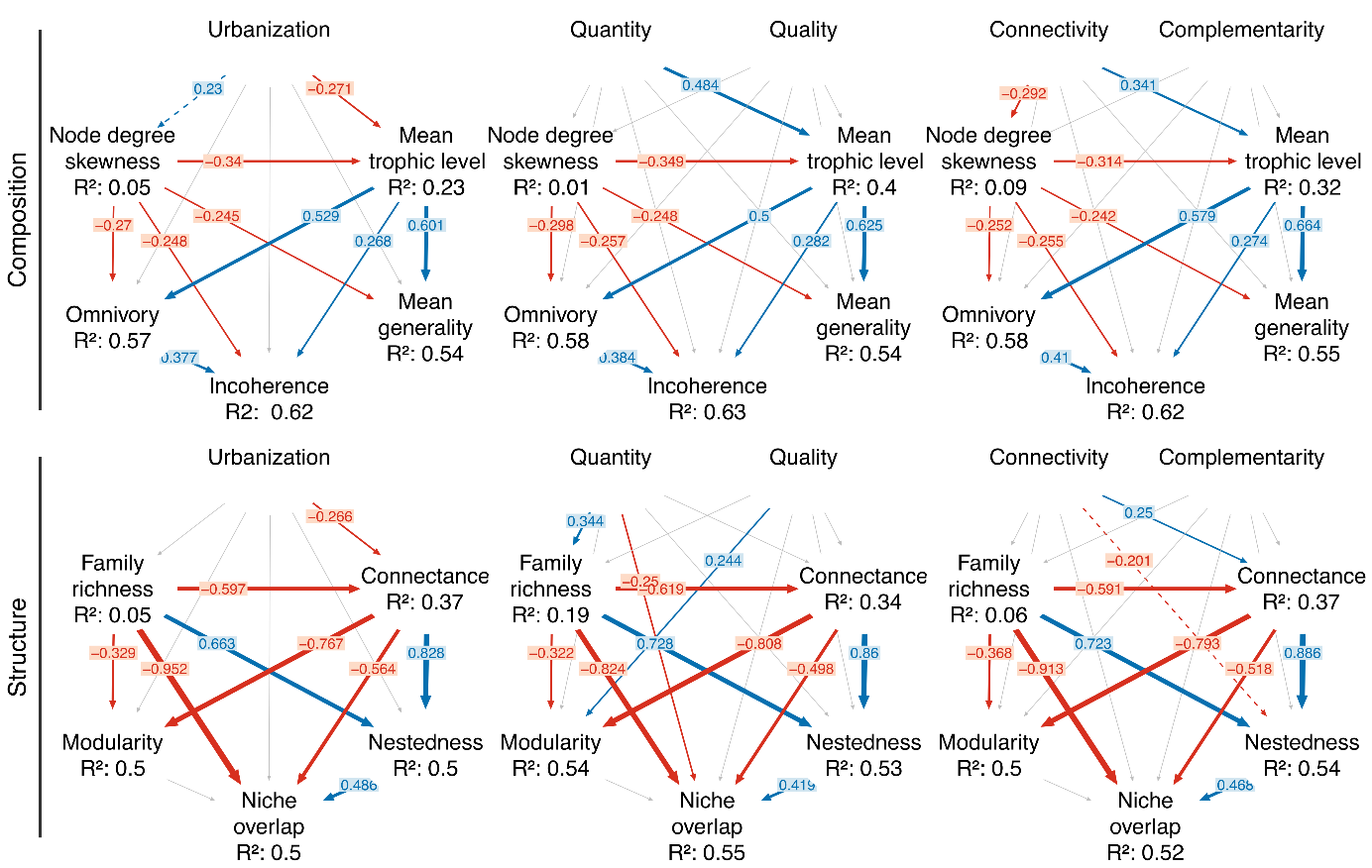
Figure S9: Structural equation models showing relationships between compositional (top) and structural (bottom) food web properties. Blue arrows represent significant positive relationships, red arrows represent significant negative relationships (alpha ≤ 0.05), and dashed arrows indicate marginally significant correlations (alpha ≤ 0.1). Arrow thickness reflects coefficient size, while grey arrows denote non-significant relationships (coefficients not shown).*

Table S1: References for all trophic interactions used to construct the metaweb. The ID number(s) of the data source(s) correspond to the ID column in the metaweb data frame, which documents all interactions between source and target taxa. The full metaweb is available on GitHub at <https://github.com/KPerrelet/Urban_food_webs>.

| ID | Full Citation |
| --- | --- |
| 1 | Poelen, J. H., Simons, J. D., & Mungall, C. J. (2014). Global biotic interactions: An open infrastructure to share and analyze species-interaction datasets. Ecological Informatics, 24, 148–159. https://doi.org/10.1016/j.ecoinf.2014.08.005 |
| 2 | Reji Chacko, M., Albouy, C., Altermatt, F., Brändle, M., Casanelles Abella, J., Boussange, V., Campell, F., Ellis, W. N., Fopp, F., Gossner, M. M., Ho., H., Joss, A., Kipf, P., Neff, F., Petrović, A., Prié, V., Tomanović, Ž., Zimmerli, N., Pellissier, L. (2024). trophiCH v1 - a food web for Switzerland. EnviDat. https://www.doi.org/10.16904/envidat.467. |
| 3 | Arachnologische Gesellschaft e. V. (n.d.). Wiki der Arachnologischen Gesellschaft e. V. Retrieved 26 June 2024, from https://wiki.arages.de/ |
| 4 | Sarma, S. S., Larios Jurado, P. S., & Nandini, S. (2001). Effect of three food types on the population growth of Brachionus calyciflorus and Brachionus patulus (Rotifera: Brachionidae). Revista De Biologia Tropical, 49(1), 77–84. |
| 5 | Candonidae. (2024). [Database]. Mindat.Org. https://www.mindat.org/taxon-5192.html |
| 6 | Damkaer, D. M. (2002). The copepodologist’s cabinet: a biographical and bibliographical history (Vol. 240). American Philosophical Society. |
| 7 | Griggs, J. A., Shiel, R. J., & Croome, R. L. (1999). Australian Chydoridae (Crustacea: Branchiopoda: Anomopoda): Taxonomic impediments. In W. Ponder & D. Lunney (Eds.), The Other 99%: The Conservation and Biodiversity of Invertebrates (p. 0). Royal Zoological Society of New South Wales. https://doi.org/10.7882/RZSNSW.1999.034 |
| 8 | Fryer, G. (1957). The Feeding Mechanism of Some Freshwater Cyclopoid Copepods. Proceedings of the Zoological Society of London, 129(1), 1–25. https://doi.org/10.1111/j.1096-3642.1957.tb00278.x |
| 9 | Centre for Freshwater Ecosystems. (n.d.). Ostracoda (seed shrimps) Podocopida. Identification and Ecology of Australian Freshwater Invertebrates. Retrieved 26 June 2024, from https://www.mdfrc.org.au/bugguide/display.asp?type=9&class=18&subclass=30&couplet=0 |
| 10 | Fryer, G. (1987). The feeding mechanisms of the Daphniidae (Crustacea: Cladocera): recent suggestions and neglected considerations. Journal of Plankton Research, 9(3), 419–432. https://doi.org/10.1093/plankt/9.3.419 |
| 11 | Todaro, M. A. (2019). Gastrotricha >> Overview. UNIVERSITY OF MODENA & REGGIO EMILIA ITALY. http://www.gastrotricha.unimore.it/overview.htm |
| 12 | Higgot, M. (n.d.). Dicyrtomidae—Globular springtails \| NatureSpot. NatureSpot Wildlife and Wild Places of Leicestershire & Rutland. Retrieved 26 June 2024, from https://www.naturespot.org.uk/family/dycyrtomidae |
| 13 | Nelson, D. (2022, July 19). Ectopsocus californicus. 10,000 Things of the Pacific Northwest. http://10000thingsofthepnw.com/2022/07/19/ectopsocus-californicus/ |
| 14 | Smithers, C. N. (2014). Family ELIPSOCIDAE. Australian Government, Department of Climate Change, Energy, the Environment and Water. https://biodiversity.org.au/afd/taxa/ELIPSOCIDAE |
| 15 | Ellobiidae. (2024). [Database]. Mindat.Org. https://www.mindat.org/taxon-9457.html |
| 16 | Gajda, Ł., Gorgoń, S., & Urbisz, A. Z. (2017). Food preferences of enchytraeids. Pedobiologia, 63, 19–36. https://doi.org/10.1016/j.pedobi.2017.06.002 |
| 17 | Bernard, E. C. (2023). Soil arthropods: Underfoot and all around. In M. J. Goss & M. Oliver (Eds.), Encyclopedia of Soils in the Environment (Second Edition) (pp. 70–104). Academic Press. https://doi.org/10.1016/B978-0-12-822974-3.00204-4 |
| 18 | Kutschera, U. (2003). The Feeding Strategies of the Leech Erpobdella octoculata (L.): A Laboratory Study. International Review of Hydrobiology, 88(1), 94–101. https://doi.org/10.1002/iroh.200390008 |
| 19 | Kotov, A. (2016). Identification Guides to the Plankton and Benthos of Inland Waters: CLADOCERA - Family Eurycercidae (Vol. 25). Margraf Publishers. |
| 20 | Hamrsky, J. (n.d.). Hydra—LIFE IN FRESHWATER. LIFE IN FRESHWATER - Macro Photography of Aquatic Insects and Other Freshwater Invertebrates. Retrieved 26 June 2024, from https://lifeinfreshwater.net/hydra/ |
| 21 | Proctor, H. C. (2009). Hydrachnida (Water Mites). In G. E. Likens (Ed.), Encyclopedia of Inland Waters (pp. 335–345). Academic Press. https://doi.org/10.1016/B978-012370626-3.00176-9 |
| 22 | Hofstetter, R. W., Dinkins-Bookwalter, J., Davis, T. S., & Klepzig, K. D. (2015). Chapter 6—Symbiotic Associations of Bark Beetles. In F. E. Vega & R. W. Hofstetter (Eds.), Bark Beetles (pp. 209–245). Academic Press. https://doi.org/10.1016/B978-0-12-417156-5.00006-X |
| 23 | Castaño-Meneses, G., Palacios-Vargas, J. G., & Cutz-Pool, L. Q. (2004). Feeding habits of Collembola and their ecological niche. Anales del Instituto de Biología. Serie Zoología, 75(1), 135–142. Redalyc. |
| 24 | Janssens, F. (2024). Checklist of the Collembola: Katiannidae [Database]. Checklist of the Collembola. http://collembola.org/taxa/katiidae.htm |
| 25 | Trombidiformes (n.d.). Wiki der Arachnologischen Gesellschaft e. V. Retrieved 26 June 2024, from https://wiki.arages.de/index.php?title=Trombidiformes |
| 26 | Museums Victoria Staff (2010) Lithobiidae Lithobiid Centipede in Museums Victoria Collections https://collections.museumsvictoria.com.au/species/8754 Accessed 27 June 2024 |
| 27 | Higgot, M. (n.d.). Lumbricidae \| NatureSpot. NatureSpot Wildlife and Wild Places of Leicestershire & Rutland. Retrieved 26 June 2024, from https://www.naturespot.org.uk/family/lumbricidae |
| 28 | Austin, A., Fagan-Jeffries, E., Harvey, M., Hodda, M., Jennings, J., Stephens, C., Yeates, D., & Volschenk, E. (2024). Family Lumbriculidae [Database]. Key to Australian Freshwater and Terrestrial Invertebrates. https://keys.lucidcentral.org/keys/v3/TFI/start%20key/key/Annelida%20key/Media/Html/Lumbriculidae.html |
| 29 | Nozaki, M., Ito, K., Miura, C., & Miura, T. (2013). Examination of Digestive Enzyme Distribution in Gut Tract and Functions of Intestinal Caecum, in Megascolecid Earthworms (Oligochaeta: Megascolecidae) in Japan. Zoological Science, 30(9), 710–715. https://doi.org/10.2108/zsj.30.710 |
| 30 | Strohmeyer, C. (2023). Aquarium, Fish Parasites, Worms; Planaria, Nematodes, Detritus, Anchor. https://www.aquarium-pond-answers.com/2007/03/trematodes-and-nematodes-in-fish.html |
| 31 | Greenslade, P., Moore, S. & Farrow, R (2002). Observations on the feeding behaviour of Uchidanurinae (Collembola: Neanuridae) in Australia. Victorian Nat. 119, 221–223. |
| 32 | Higgot, M. (n.d.).Neelidae \| NatureSpot. NatureSpot Wildlife and Wild Places of Leicestershire & Rutland. Retrieved 26 June 2024, from https://www.naturespot.org.uk/family/neelidae |
| 33 | Hodkinson, I. D., Coulson, S., Webb, N. R., Block, W., Strathdee, A. T., & Bale, J. S. (1994). Feeding studies on Onychiurus arcticus (Tullberg) (Collembola: Onychiuridae) on West Spitsbergen. Polar Biology, 14(1), 17–19. https://doi.org/10.1007/BF00240267 |
| 34 | Santamaria, B., Verbeken, A., & Haelewaters, D. (2023). Mycophagy: A Global Review of Interactions between Invertebrates and Fungi. Journal of Fungi, 9(2). https://doi.org/10.3390/jof9020163 |
| 35 | Szczepanska, A., and W. Magowski. 2006. Four species of the podapolipid mites (Acari: Podapolipidae parasitising the carabid beetles (Coleoptera: Carabidae) new to the fauna of Poland. Pol. J. Entomol. 75:491–497 |
| 36 | Krivosheina, N. P. (2011). New data on the larval morphology of limoniid flies of the genus Ula (Diptera, Pediciidae). Entomological Review, 91(4), 432–443. https://doi.org/10.1134/S001387381104004X |
| 37 | Anonby, J. E. (2019). Psocoptera of Canada. ZooKeys, 819, 295–299. https://doi.org/10.3897/zookeys.819.27640 |
| 38 | Allard, C. M., & Yeargan, K. V. (2005). Effect of Diet on Development and Reproduction of the Harvestman Phalangium opilio (Opiliones: Phalangiidae). Environmental Entomology, 34(1), 6–13. https://doi.org/10.1603/0046-225X-34.1.6 |
| 39 | Schmaedick, M. (n.d.). Phalangium opilio. Cornell University College of Agriculture and Life Sciences. Retrieved 26 June 2024, from https://biocontrol.entomology.cornell.edu/predators/Phalangium.php |
| 40 | Philoscia muscorum—Facts, Diet, Habitat & Pictures on Animalia.bio. (n.d.). Animalia. Retrieved 26 June 2024, from https://animalia.bio/index.php/philoscia-muscorum |
| 41 | Jewiss-Gaines, A., Marshall, S. A., & Whitworth, T., L. (2012). Cluster Flies (Calliphoridae: Polleniinae: Pollenia) of North America. Canadian Journal of Arthropod Identification, 19. https://doi.org/10.3752/cjai.2012.19 |
| 42 | Lee, D. C. (1974). Rhodacaridae acari mesostigmata from near adelaide australia part 3 behavior and development. Acarologia, 16(1), 21–44. |
| 43 | Hackton, M. (n.d.). SCRAPTIIDAE. Uk Beetles. Retrieved 26 June 2024, from https://www.ukbeetles.co.uk/scraptiidae |
| 44 | Higgot, M. (n.d.). Scutigerella agg. \| NatureSpot. NatureSpot Wildlife and Wild Places of Leicestershire & Rutland. Retrieved 26 June 2024, from https://www.naturespot.org.uk/species/scutigerella-immaculata |
| 45 | Pfingstl, T., & Krisper, G. (2011). No difference in the juveniles of two Tectocepheus species (Acari: Oribatida, Tectocepheidae). Acarologia, 51(2), Article 2. https://doi.org/10.1051/acarologia/20112005 |
| 46 | Gerlach, A., Russell, D. J., Jaeschke, B., & Römbke, J. (2014). Feeding preferences of native terrestrial isopod species (Oniscoidea, Isopoda) for native and introduced leaf litter. Applied Soil Ecology, 83, 95–100. https://doi.org/10.1016/j.apsoil.2014.02.006 |
| 47 | Kautz, G., Zimmer, M., & Topp, W. (2000). Responses of the parthenogenetic isopod, Trichoniscus pusillus (Isopoda: Oniscidea), to changes in food quality. Pedobiologia, 44(1), 75–85. https://doi.org/10.1078/S0031-4056(04)70029-3 |
| 48 | Nagel, L., Zanuttig, M., & Forbes, M. R. (2011). Escape of parasitic water mites from dragonfly predators attacking their damselfly hosts. Canadian Journal of Zoology, 89(3), 213–218. https://doi.org/10.1139/Z10-112 |
| 49 | Yin, X., Jin, W., Zhou, Y., Wang, P., & Zhao, W. (2017). Hidden defensive morphology in rotifers: Benefits, costs, and fitness consequences. Scientific Reports, 7(1), 4488. https://doi.org/10.1038/s41598-017-04809-z |
| 50 | Vesterinen, E. J., Kaunisto, K. M., & Lilley, T. M. (2020). A global class reunion with multiple groups feasting on the declining insect smorgasbord. Scientific Reports, 10(1), 16595. https://doi.org/10.1038/s41598-020-73609-9 |
| 51 | Smith, R. J. (2024). Predation on Ostracods. Ostracode Research at the Lake Biwa Museum. https://www.biwahaku.jp/smith/ostracod_predation.html |
| 52 | Woodward, G., & Hildrew, A. G. (2001). Invasion of a stream food web by a new top predator. Journal of Animal Ecology, 70(2), 273–288. https://doi.org/10.1111/j.1365-2656.2001.00497.x |
| 53 | Musser, F. R., & Shelton, A. M. (2003). Predation of Ostrinia nubilalis (Lepidoptera: Crambidae) Eggs in Sweet Corn by Generalist Predators and the Impact of Alternative Foods. Environmental Entomology, 32(5), 1131–1138. https://doi.org/10.1603/0046-225X-32.5.1131 |
| 54 | Adams, J. B., Bollens, S. M., & Bishop, J. G. (2015). Predation on the Invasive Copepod, Pseudodiaptomus forbesi, and Native Zooplankton in the Lower Columbia River: An Experimental Approach to Quantify Differences in Prey-Specific Feeding Rates. PLoS ONE, 10(11), e0144095. https://doi.org/10.1371/journal.pone.0144095 |
| 55 | Horstmann, M., Tollrian, R., & Weiss, L. C. (2021). Thwarting predators? A three-dimensional perspective of morphological alterations in the freshwater crustacean Daphnia. PLoS ONE, 16(7), e0254263. https://doi.org/10.1371/journal.pone.0254263 |
| 57 | Wright, J. (2014). Gastrotricha (hairy back worms). Animal Diversity Web. https://animaldiversity.org/accounts/Gastrotricha/ |
| 58 | Koehler, H. H. (1999). Predatory mites (Gamasina, Mesostigmata). In M. G. Paoletti (Ed.), Invertebrate Biodiversity as Bioindicators of Sustainable Landscapes (pp. 395–410). Elsevier. https://doi.org/10.1016/B978-0-444-50019-9.50022-4 |
| 59 | Forest Cockroach (Ectobius sylvestris). (n.d.). Picture Insect. Retrieved 26 June 2024, from https://pictureinsect.com/wiki/Ectobius_sylvestris.html |
| 60 | Smithsonian Institution. (n.d.). Wolf Spider. Smithsonian Institution. Retrieved 26 June 2024, from https://www.si.edu/newsdesk/snapshot/wolf-spider |
| 61 | Suvak, M. (2011). Predatory and parasitic insects in greenhouses of Botanical Garden of P.J.Šafárik University in Košice, Slovakia. Thaiszia Journal of Botany, 21, 185–205. |
| 63 | Thornton, I. W. B. (1985). The Geographical and Ecological Distribution of Arboreal Psocoptera. Annual Review of Entomology, 30(Volume 30, 1985), 175–196. https://doi.org/10.1146/annurev.en.30.010185.001135 |
| 64 | Schmelz, R. M. (2001). Enchytraeidae as prey of Dolichopodidae, recent and in Baltic amber (Oligochaeta; Diptera). https://www.zobodat.at/pdf/Bonner-Zoologische-Beitraege_50_0089-0101.pdf |
| 65 | Bernard, E. C. (2023). Soil arthropods: Underfoot and all around. In M. J. Goss & M. Oliver (Eds.), Encyclopedia of Soils in the Environment (Second Edition) (pp. 70–104). Academic Press. https://doi.org/10.1016/B978-0-12-822974-3.00204-4 |
| 68 | Murillo, A. C., Hubbard, C. B., Hinkle, N. C., & Gerry, A. C. (2021). Big Problems With Little House Fly (Diptera: Fanniidae). Journal of Integrated Pest Management, 12(1), 40. https://doi.org/10.1093/jipm/pmaa023 |
| 69 | Lacewings \| University of Maryland Extension. (2023). [University of Michigan]. https://extension.umd.edu/resource/lacewings |
| 70 | Turner, J. R. G. (2013). The dawn flight of the gold swift Hepialus hecta: Predator avoidance and the integration of complex lek behaviour (Lepidoptera, Hepialidae). Biological Journal of the Linnean Society, 110(2), 305–319. https://doi.org/10.1111/bij.12145 |
| 72 | Hydra. (n.d.). Aquasabi. Retrieved 26 June 2024, from https://www.aquasabi.com/aquascaping-wiki_parasites_hydra |
| 73 | Lindsey, J. K. (2013). Hypogastruridae. The Ecology of Commanster. https://www.commanster.eu/Commanster/Invertebrates/Collembola/Hypogastruridae.html |
| 74 | Lindsey, J. K. (2010). Isotomidae. The Ecology of Commanster. https://www.commanster.eu/Commanster/Invertebrates/Collembola/Isotomidae.html |
| 75 | Eitzinger, B., Abrego, N., Gravel, D., Huotari, T., Vesterinen, E. J., & Roslin, T. (2019). Assessing changes in arthropod predator–prey interactions through DNA-based gut content analysis—Variable environment, stable diet. Molecular Ecology, 28(2), 266–280. https://doi.org/10.1111/mec.14872 |
| 76 | Tricholauxania praeusta (n.d.). Picture Insect. Retrieved 26 June 2024, from https://pictureinsect.com/wiki/Tricholauxania_praeusta.html |
| 77 | Costa, B. G., Pellegrini, T. G., Bernardi, L. F. de O., & Ferreira, R. L. (2017). Notes on predator-prey relationships among Tanypodinae larvae (Diptera, Chironomidae) and mites (Acariformes) in Brazilian subterranean aquatic environments. Subterranean Biology, 22, 67–74. https://doi.org/10.3897/subtbiol.22.13925 |
| 79 | Lonchoptera bifurcata (n.d.). Picture Insect. Retrieved 26 June 2024, from https://pictureinsect.com/wiki/Lonchoptera_bifurcata.html |
| 80 | Lindsey, J. K. (2005). Lumbricidae. The Ecology of Commanster. https://www.commanster.eu/Commanster/Invertebrates/Collembola/Lumbricidae.html |
| 81 | Head, S. (n.d.). Pond worms. Retrieved 26 June 2024, from http://www.wlgf.org/wildlife/pond_worms |
| 84 | Lindsey, J. K. (2011). Neanuridae. The Ecology of Commanster. https://www.commanster.eu/Commanster/Invertebrates/Collembola/Neanuridae.html |
| 85 | Groth, E.M. (1997) [Thesis] Ecology of the Predatory Mite, Pergamasus quisquiliarum Canestrini (Acari: Mesostigmata). https://www.google.com/url?sa=t&source=web&rct=j&opi=89978449&url=https://ir.library.oregonstate.edu/downloads/dz010t216&ved=2ahUKEwiDr5D3y8mGAxX8_QIHHcEuAYsQFnoECBUQAQ&usg=AOvVaw07m_uz2eRcpQ7mvhnm6uQg |
| 87 | Greenslade, P., & Ireson, J. (2022). Onychiuridae (Collembola) of Australia: A key to species with notes on their distributions and pest status. Austral Entomology, 61(2), 187–198. https://doi.org/10.1111/aen.12594 |
| 88 | Tamaddoni-Nezhad, A., Milani, G. A., Raybould, A., Muggleton, S., & Bohan, D. A. (2013). Chapter Four—Construction and Validation of Food Webs Using Logic-Based Machine Learning and Text Mining. In G. Woodward & D. A. Bohan (Eds.), Advances in Ecological Research (Vol. 49, pp. 225–289). Academic Press. https://doi.org/10.1016/B978-0-12-420002-9.00004-4 |
| 89 | Baz, A. (2004). Bark-Lice, Book-Lice or Psocids (Psocoptera). In Encyclopedia of Entomology (pp. 236–250). Kluwer Academic Publishers. https://doi.org/10.1007/0-306-48380-7_416 |
| 90 | British Arachnological Society. (2023, January 31). Spiders and harvestmen \| NatureScot. https://www.nature.scot/plants-animals-and-fungi/invertebrates/land-invertebrates/spiders-and-harvestmen |
| 91 | Philosciamuscorum (n.d.). Picture Insect. Retrieved 26 June 2024, from https://pictureinsect.com/wiki/Philoscia_muscorum.html |
| 93 | Utah State University. (n.d.). Sympylans (Garden Centipede) \| USU. Retrieved 26 June 2024, from https://extension.usu.edu/vegetableguide/leafy-greens/garden-centipede |
| 94 | Pont, & Meier, R. (2002). The Sepsidae (Diptera) of Europe. BRILL. https://doi.org/10.1163/9789047401391 |
| 96 | Verschut, V., Strandmark, A., Esparza-Salas, R., & Hambäck, P. A. (2019). Seasonally varying marine influences on the coastal ecosystem detected through molecular gut analysis. Molecular Ecology, 28(2), 307–317. https://doi.org/10.1111/mec.14830 |
| 98 | Anthony, D. W. (n.d.). XI. PATHOGENS OF TABANIDAE (HORSEFLIES). https://iris.who.int/bitstream/handle/10665/262034/PMC2395947.pdf?sequence=1&isAllowed=y |
| 99 | Jałoszyński, P., & Olszanowski, Z. (2015). Feeding of Scydmaenus rufus (Coleoptera: Staphylinidae: Scydmaeninae) on oribatid and uropodine mites: Prey preferences and hunting behaviour. EJE, 112(1), 151–164. https://doi.org/10.14411/eje.2015.023 |
| 101 | USMANTIS. (n.d.). Isopods Trichoniscidae sp. “Dwarf Purple” bio-active cleanup crew. USMANTIS. Retrieved 26 June 2024, from https://usmantis.com/products/isopods-trichoniscidae-sp-dwarf-purple |
| 102 | Parimuchová, A., Dušátková, L. P., Kováč, Ľ., Macháčková, T., Slabý, O., & Pekár, S. (2021). The food web in a subterranean ecosystem is driven by intraguild predation. Scientific Reports, 11(1), 4994. https://doi.org/10.1038/s41598-021-84521-1 |
| 103 | Leung, R., Moisset, B., Gruber, J., & Sciurorum, V. (2017). Family Sphaeroceridae—Lesser Dung Flies. Bugguide. https://bugguide.net/node/view/17765 |

Table S2: Goodness-of-fit statistics for the structural equation models (SEM) assessing compositional and structural food web properties. Fit was evaluated using Fisher’s C and Chi-squared tests, with corresponding coefficients, degrees of freedom, and p-values reported for each model..

| SEM type | Land-use type tested | Test | Coefficient | Degrees of freedom | P-value |
| --- | --- | --- | --- | --- | --- |
| Composition | Urbanization | Chi-Squared | 3.571 | 2 | 0.168 |
|  |  | Fisher's C | 6.11 | 4 | 0.191 |
|  | Quantity -  quality | Chi-Squared | 3.751 | 2 | 0.153 |
|  |  | Fisher's C | 6.393 | 4 | 0.172 |
|  | Connectivity -  complementarity | Chi-Squared | 4.387 | 2 | 0.112 |
|  |  | Fisher's C | 7.028 | 4 | 0.134 |
| Structure | Urbanization | Chi-Squared | 0.318 | 1 | 0.573 |
|  |  | Fisher's C | 1.044 | 2 | 0.593 |
|  | Quantity -  quality | Chi-Squared | 0.432 | 1 | 0.511 |
|  |  | Fisher's C | 1.241 | 2 | 0.538 |
|  | Connectivity -  complementarity | Chi-Squared | 0.518 | 1 | 0.472 |
|  |  | Fisher's C | 1.388 | 2 | 0.5 |

Table S3: Independence claims tested within the structural equation models (SEMs) for compositional and structural food web properties. Each claim corresponds to a hypothesized absence of a direct relationship between variables, as implied by the model structure. For each model, the associated degrees of freedom, critical value, and p-value are reported, indicating whether the independence assumption holds based on standard significance thresholds.

| SEM type | Land-use type tested | Independence claims | Degrees of freedom | Critical value | P-value |
| --- | --- | --- | --- | --- | --- |
| Composition | Urbanization | Omnivory ~ Mean generality | 49 | -1.644 | 0.107 |
|  |  | Coherence ~ Mean generality | 48 | 0.775 | 0.442 |
|  | Quantity -  quality | Omnivory ~ Mean generality | 48 | -1.458 | 0.151 |
|  |  | Coherence ~ Mean generality | 47 | 1.116 | 0.27 |
|  | Connectivity -  complementarity | Omnivory ~ Mean generality | 48 | -1.762 | 0.085 |
|  |  | Coherence ~ Mean generality | 47 | 0.939 | 0.352 |
| Structure | Urbanization | Nestedness ~ Modularity | 49 | -0.538 | 0.593 |
|  | Quantity -  quality | Nestedness ~ Modularity | 48 | -0.621 | 0.538 |
|  | Connectivity -  complementarity | Nestedness ~ Modularity | 48 | -0.68 | 0.5 |

Table S4: Coefficient summary of the structural equation models (SEMs) for compositional and structural food web properties. The table reports, for each path in the model:estimate (unstandardized coefficient), standard error, degrees of freedom, critical value, p-value, and standardized estimate. Paths include both relationships among food web properties and between food web properties and land-use variables.

| SEM type | Land-use type tested | Response | Predictor | Estimate | Standard error | Degrees of freedom | Critical value | P-value | Standardized estimate |
| --- | --- | --- | --- | --- | --- | --- | --- | --- | --- |
| Composition | Urbanization | Node degree skewness | Urbanization | 0.042 | 0.024 | 52 | 1.707 | 0.094 | 0.23 |
|  |  | Mean trophic level | Node degree skewness | -0.088 | 0.033 | 51 | -2.693 | 0.01 | -0.34 |
|  |  | Mean trophic level | Urbanization | -0.013 | 0.006 | 51 | -2.15 | 0.036 | -0.271 |
|  |  | Mean generality | Node degree skewness | -1.12 | 0.469 | 50 | -2.39 | 0.021 | -0.245 |
|  |  | Mean generality | Mean trophic level | 10.619 | 1.878 | 50 | 5.656 | 0 | 0.601 |
|  |  | Mean generality | Urbanization | -0.037 | 0.083 | 50 | -0.452 | 0.654 | -0.045 |
|  |  | Omnivory | Node degree skewness | -0.032 | 0.012 | 50 | -2.58 | 0.013 | -0.271 |
|  |  | Omnivory | Mean trophic level | 0.24 | 0.049 | 50 | 4.856 | 0 | 0.529 |
|  |  | Omnivory | Urbanization | -0.003 | 0.002 | 50 | -1.214 | 0.231 | -0.124 |
|  |  | Incoherence | Node degree skewness | -0.031 | 0.013 | 49 | -2.416 | 0.02 | -0.248 |
|  |  | Incoherence | Mean trophic level | 0.127 | 0.058 | 49 | 2.208 | 0.032 | 0.268 |
|  |  | Incoherence | Omnivory | 0.395 | 0.136 | 49 | 2.905 | 0.006 | 0.377 |
|  |  | Incoherence | Urbanization | -0.002 | 0.002 | 49 | -0.968 | 0.338 | -0.092 |
|  | Quantity - quality | Node degree skewness | Quantity | -0.019 | 0.025 | 51 | -0.744 | 0.46 | -0.104 |
|  |  | Node degree skewness | Quality | -0.004 | 0.025 | 51 | -0.153 | 0.879 | -0.021 |
|  |  | Mean trophic level | Node degree skewness | -0.091 | 0.029 | 50 | -3.178 | 0.003 | -0.349 |
|  |  | Mean trophic level | Quantity | 0.023 | 0.005 | 50 | 4.399 | 0 | 0.484 |
|  |  | Mean trophic level | Quality | 0.003 | 0.005 | 50 | 0.654 | 0.516 | 0.072 |
|  |  | Mean generality | Node degree skewness | -1.135 | 0.47 | 49 | -2.418 | 0.019 | -0.248 |
|  |  | Mean generality | Mean trophic level | 11.042 | 2.126 | 49 | 5.194 | 0 | 0.625 |
|  |  | Mean generality | Quantity | 0.006 | 0.091 | 49 | 0.063 | 0.95 | 0.007 |
|  |  | Mean generality | Quality | -0.093 | 0.077 | 49 | -1.207 | 0.233 | -0.113 |
|  |  | Omnivory | Node degree skewness | -0.035 | 0.013 | 49 | -2.798 | 0.007 | -0.298 |
|  |  | Omnivory | Mean trophic level | 0.226 | 0.057 | 49 | 3.991 | 0 | 0.5 |
|  |  | Omnivory | Quantity | 0.002 | 0.002 | 49 | 0.904 | 0.37 | 0.104 |
|  |  | Omnivory | Quality | 0.001 | 0.002 | 49 | 0.606 | 0.547 | 0.059 |
|  |  | Incoherence | Node degree skewness | -0.032 | 0.013 | 48 | -2.468 | 0.017 | -0.257 |
|  |  | Incoherence | Mean trophic level | 0.134 | 0.062 | 48 | 2.164 | 0.035 | 0.283 |
|  |  | Incoherence | Omnivory | 0.403 | 0.136 | 48 | 2.97 | 0.005 | 0.384 |
|  |  | Incoherence | Quantity | 0 | 0.002 | 48 | -0.102 | 0.919 | -0.011 |
|  |  | Incoherence | Quality | 0.003 | 0.002 | 48 | 1.414 | 0.164 | 0.125 |
|  | Connectivity - complementarity | Node degree skewness | Connectivity | -0.053 | 0.024 | 51 | -2.168 | 0.035 | -0.292 |
|  |  | Node degree skewness | Complementarity | 0.013 | 0.024 | 51 | 0.542 | 0.59 | 0.073 |
|  |  | Mean trophic level | Node degree skewness | -0.081 | 0.032 | 50 | -2.57 | 0.013 | -0.314 |
|  |  | Mean trophic level | Connectivity | 0.016 | 0.006 | 50 | 2.781 | 0.008 | 0.341 |
|  |  | Mean trophic level | Complementarity | 0.009 | 0.006 | 50 | 1.617 | 0.112 | 0.19 |
|  |  | Mean generality | Node degree skewness | -1.106 | 0.474 | 49 | -2.332 | 0.024 | -0.242 |
|  |  | Mean generality | Mean trophic level | 11.73 | 1.992 | 49 | 5.889 | 0 | 0.664 |
|  |  | Mean generality | Connectivity | -0.047 | 0.087 | 49 | -0.542 | 0.591 | -0.057 |
|  |  | Mean generality | Complementarity | -0.08 | 0.079 | 49 | -1.009 | 0.318 | -0.097 |
|  |  | Omnivory | Node degree skewness | -0.03 | 0.013 | 49 | -2.38 | 0.021 | -0.252 |
|  |  | Omnivory | Mean trophic level | 0.262 | 0.052 | 49 | 5.011 | 0 | 0.579 |
|  |  | Omnivory | Connectivity | 0.002 | 0.002 | 49 | 0.654 | 0.516 | 0.07 |
|  |  | Omnivory | Complementarity | -0.003 | 0.002 | 49 | -1.496 | 0.141 | -0.148 |
|  |  | Incoherence | Node degree skewness | -0.031 | 0.013 | 48 | -2.43 | 0.019 | -0.255 |
|  |  | Incoherence | Mean trophic level | 0.13 | 0.063 | 48 | 2.057 | 0.045 | 0.274 |
|  |  | Incoherence | Omnivory | 0.429 | 0.14 | 48 | 3.063 | 0.004 | 0.41 |
|  |  | Incoherence | Connectivity | 0 | 0.002 | 48 | -0.129 | 0.898 | -0.013 |
|  |  | Incoherence | Complementarity | 0.001 | 0.002 | 48 | 0.357 | 0.723 | 0.034 |
| Structure | Urbanization | Taxonomic richness | Urbanization | -0.019 | 0.014 | 52 | -1.335 | 0.182 | -0.169 |
|  |  | Connectance | Urbanization | -0.002 | 0.001 | 51 | -2.363 | 0.022 | -0.266 |
|  |  | Connectance | Taxonomic richness | -0.001 | 0 | 51 | -5.309 | 0 | -0.597 |
|  |  | Modularity | Urbanization | 0.002 | 0.002 | 50 | 1.423 | 0.161 | 0.153 |
|  |  | Modularity | Taxonomic richness | -0.001 | 0 | 50 | -2.592 | 0.013 | -0.329 |
|  |  | Modularity | Connectance | -1.319 | 0.218 | 50 | -6.059 | 0 | -0.767 |
|  |  | Nestedness | Urbanization | 0.042 | 0.145 | 50 | 0.289 | 0.774 | 0.031 |
|  |  | Nestedness | Taxonomic richness | 0.086 | 0.016 | 50 | 5.267 | 0 | 0.663 |
|  |  | Nestedness | Connectance | 125.586 | 19.061 | 50 | 6.589 | 0 | 0.828 |
|  |  | Niche overlap | Nestedness | 0.003 | 0.001 | 48 | 3.342 | 0.002 | 0.486 |
|  |  | Niche overlap | Modularity | 0.074 | 0.064 | 48 | 1.144 | 0.258 | 0.165 |
|  |  | Niche overlap | Urbanization | 0 | 0.001 | 48 | 0.548 | 0.586 | 0.061 |
|  |  | Niche overlap | Taxonomic richness | -0.001 | 0 | 48 | -5.743 | 0 | -0.952 |
|  |  | Niche overlap | Connectance | -0.432 | 0.156 | 48 | -2.774 | 0.008 | -0.564 |
|  | Quantity - quality | Taxonomic richness | Quantity | 0.038 | 0.014 | 51 | 2.694 | 0.007 | 0.344 |
|  |  | Taxonomic richness | Quality | -0.012 | 0.014 | 51 | -0.812 | 0.417 | -0.106 |
|  |  | Connectance | Quantity | 0.002 | 0.001 | 50 | 1.645 | 0.106 | 0.202 |
|  |  | Connectance | Quality | 0 | 0.001 | 50 | 0.128 | 0.899 | 0.015 |
|  |  | Connectance | Taxonomic richness | -0.001 | 0 | 50 | -5.047 | 0 | -0.619 |
|  |  | Modularity | Quantity | -0.002 | 0.002 | 49 | -1.005 | 0.32 | -0.107 |
|  |  | Modularity | Quality | 0.004 | 0.002 | 49 | 2.502 | 0.016 | 0.244 |
|  |  | Modularity | Taxonomic richness | -0.001 | 0 | 49 | -2.537 | 0.014 | -0.322 |
|  |  | Modularity | Connectance | -1.39 | 0.205 | 49 | -6.778 | 0 | -0.809 |
|  |  | Nestedness | Quantity | -0.22 | 0.147 | 49 | -1.499 | 0.14 | -0.162 |
|  |  | Nestedness | Quality | -0.021 | 0.135 | 49 | -0.152 | 0.88 | -0.015 |
|  |  | Nestedness | Taxonomic richness | 0.095 | 0.017 | 49 | 5.643 | 0 | 0.728 |
|  |  | Nestedness | Connectance | 130.321 | 18.367 | 49 | 7.095 | 0 | 0.86 |
|  |  | Niche overlap | Nestedness | 0.002 | 0.001 | 47 | 2.925 | 0.005 | 0.419 |
|  |  | Niche overlap | Modularity | 0.058 | 0.065 | 47 | 0.89 | 0.378 | 0.13 |
|  |  | Niche overlap | Quantity | -0.002 | 0.001 | 47 | -2.241 | 0.03 | -0.25 |
|  |  | Niche overlap | Quality | 0 | 0.001 | 47 | 0.302 | 0.764 | 0.032 |
|  |  | Niche overlap | Taxonomic richness | -0.001 | 0 | 47 | -4.849 | 0 | -0.824 |
|  |  | Niche overlap | Connectance | -0.382 | 0.155 | 47 | -2.457 | 0.018 | -0.498 |
|  | Connectivity - complementarity | Taxonomic richness | Connectivity | 0.018 | 0.014 | 51 | 1.264 | 0.206 | 0.162 |
|  |  | Taxonomic richness | Complementarity | 0.008 | 0.014 | 51 | 0.571 | 0.568 | 0.073 |
|  |  | Connectance | Connectivity | 0.002 | 0.001 | 50 | 2.184 | 0.034 | 0.251 |
|  |  | Connectance | Complementarity | 0 | 0.001 | 50 | -0.378 | 0.707 | -0.043 |
|  |  | Connectance | Taxonomic richness | -0.001 | 0 | 50 | -5.153 | 0 | -0.591 |
|  |  | Modularity | Connectivity | -0.001 | 0.002 | 49 | -0.729 | 0.47 | -0.078 |
|  |  | Modularity | Complementarity | 0.002 | 0.002 | 49 | 1.394 | 0.17 | 0.142 |
|  |  | Modularity | Taxonomic richness | -0.001 | 0 | 49 | -2.894 | 0.006 | -0.368 |
|  |  | Modularity | Connectance | -1.363 | 0.218 | 49 | -6.251 | 0 | -0.793 |
|  |  | Nestedness | Connectivity | -0.274 | 0.141 | 49 | -1.946 | 0.057 | -0.201 |
|  |  | Nestedness | Complementarity | 0.014 | 0.133 | 49 | 0.103 | 0.918 | 0.01 |
|  |  | Nestedness | Taxonomic richness | 0.094 | 0.016 | 49 | 5.923 | 0 | 0.723 |
|  |  | Nestedness | Connectance | 134.42 | 18.453 | 49 | 7.284 | 0 | 0.887 |
|  |  | Niche overlap | Nestedness | 0.002 | 0.001 | 47 | 3.135 | 0.003 | 0.468 |
|  |  | Niche overlap | Modularity | 0.094 | 0.064 | 47 | 1.464 | 0.15 | 0.21 |
|  |  | Niche overlap | Connectivity | 0 | 0.001 | 47 | -0.541 | 0.591 | -0.061 |
|  |  | Niche overlap | Complementarity | -0.001 | 0.001 | 47 | -1.395 | 0.17 | -0.145 |
|  |  | Niche overlap | Taxonomic richness | -0.001 | 0 | 47 | -5.325 | 0 | -0.913 |
|  |  | Niche overlap | Connectance | -0.397 | 0.16 | 47 | -2.487 | 0.017 | -0.519 |

**References**

Fournier, B., Frey, D., & Moretti, M. (2020). The origin of urban communities: From the regional species pool to community assemblages in city. *Journal of Biogeography*, *47*(3), 615–629. https://doi.org/10.1111/jbi.13772

Ho, H.-C., Brodersen, J., Gossner, M. M., Graham, C. H., Kaeser, S., Reji Chacko, M., Seehausen, O., Zimmermann, N. E., Pellissier, L., & Altermatt, F. (2022). Blue and green food webs respond differently to elevation and land use. *Nature Communications*, *13*(1), 6415. https://doi.org/10.1038/s41467-022-34132-9

Johnson, S., Domínguez-García, V., Donetti, L., & Muñoz, M. A. (2014). Trophic coherence determines food-web stability. *Proceedings of the National Academy of Sciences*, *111*(50), 17923–17928. https://doi.org/10.1073/pnas.1409077111

Reji Chacko, M., Albouy, C., Altermatt, F., Abella, J. C., Brändle, M., Boussange, V., Campell, F., Ellis, W. N., Fopp, F., Gossner, M., Ho, H.-C., Joss, A., Kipf, P., Neff, F., Petrović, A., Prié, V., Tomanović, Ž., Zimmerli, N., & Pellissier, L. (2024). *trophiCH - a national species-level trophic metaweb of 23k species for Switzerland*. https://ecoevorxiv.org/repository/view/7294/

Turnbull, L. A., Levine, J. M., Loreau, M., & Hector, A. (2013). Coexistence, niches and biodiversity effects on ecosystem functioning. *Ecology Letters*, *16*(s1), 116–127. https://doi.org/10.1111/ele.12056
